# Two-sample Mendelian randomization study of gut microbiota and inflammatory proteins: Predictive, preventive, and personalized treatment for migraine

**DOI:** 10.4103/NRR.NRR-D-25-01711

**Published:** 2026-02-05

**Authors:** Ruolan Ma, Haiyan An, Yi Feng

**Affiliations:** Department of Anesthesiology, Peking University People’s Hospital, The Second Clinical College of Peking University, Beijing, China

**Keywords:** causality, cytokines, gut–brain axis, human gut microbiota, inflammation protein, mediating proportion, mediation, mendelian randomization, microbiome, migraine

## Abstract

The human gut microbiota is increasingly recognized as a significant factor in the pathogenesis of migraine, potentially via inflammatory pathways. Identifying specific human gut microbiota components associated with migraines, along with the investigation of particular inflammatory proteins, is essential for advancing primary prediction, targeted prevention, and personalized treatment strategies for migraines. We conducted a two-sample Mendelian randomization study using publicly available summary statistics from genome-wide association studies. Data for 473 human gut microbiota taxa were obtained from the Finnish national health survey conducted by the National Institute for Health and Welfare study (FINRISK, *n* = 5959 European participants). Genome-wide association study data (https://www.ebi.ac.uk/gwas/) for 91 circulating inflammatory proteins were obtained from 14,824 participants across 11 cohorts using the Olink Target 96 Inflammation panel. Migraine outcome data were obtained from the FinnGen R12 release, with cases defined using ICD-10 code G43. All genome-wide association study analyses were adjusted for sex, age, genotyping batch, and 10 genetic principal components to control population stratification (genomic inflation factors: 1.00–1.05). Inverse variance-weighted Mendelian randomization was the primary analysis method, with Mendelian randomization-Egger, weighted median, and mode-based methods as sensitivity analyses. Two-step Mendelian randomization mediation analysis quantified the proportion of the effects of human gut microbiota on migraine that are mediated through inflammatory proteins. Thirty-seven bacterial genera were found to be associated with migraine using the inverse variance-weighted method. Of these, 18 genera exhibited a negative association, while 19 genera demonstrated a positive association with migraine risk. Additionally, eight inflammatory proteins were found to increase the risk of migraine. Among human gut microbiota, four were observed to reduce inflammatory protein levels, whereas another four were associated with increased inflammatory protein levels. Additionally, five gut microbiota were identified to influence migraine through inflammatory proteins in both Mendelian randomization analyses. Specifically, *Actinobacteria*, *Brachyspiraceae*, CAG-269 sp001915995, and *Paraglaciecola* were found to affect migraine outcomes via inflammatory proteins, with mediation proportions of 12%, 19%, 15.5%, and 6.7%, respectively. *Lawsonibacter* sp002161175 was identified to influence migraine risk through Oncostatin-M and SLAM, with mediation proportions of 15.6% and 11.3%, respectively. Our study elucidated the role of specific human gut microbiota alterations in the pathogenesis of migraine and highlighted the mediating effects of inflammatory proteins. Targeting these particular human gut microbiota alterations offers a promising strategy for predictive, preventive, and personalized medicine in migraine management, resulting in substantial clinical advancements.

## Graphical abstract

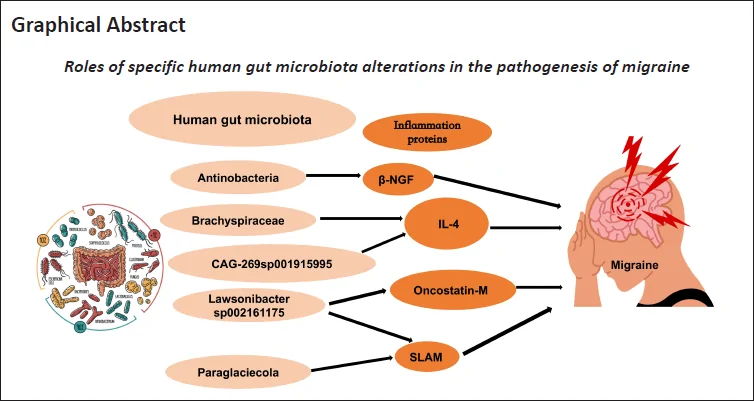

## Introduction

Migraine is a widespread neurological disorder with profound implications for individuals globally, affecting over one billion people each year (Steiner and Stovner, 2023). It transcends the simplistic characterization of a mere headache, representing instead a multifaceted condition associated with considerable economic and social burdens. Despite evidence for the role of pathophysiology, genetics, and neural networks in migraine, the exact mechanisms underlying this condition remain inadequately understood (Zhang et al., 2025). Current management strategies for migraine include the administration of acetaminophen, nonsteroidal anti-inflammatory drugs, triptans, ergot alkaloids, antiemetics, and novel therapeutic agents such as gepants (Konstantinos et al., 2020; Steiner and Stovner, 2023). Nevertheless, a universally effective treatment for migraines has yet to be identified.

The human gut microbiota (HGM), a diverse assemblage of microorganisms inhabiting the gastrointestinal tract, plays an essential role in maintaining intestinal homeostasis and modulating the host immune system (Frost et al., 2024). Recent research has elucidated the role of the HGM in the pathogenesis of migraine, emphasizing its impact on the gut–brain axis (Patel et al., 2025), the gut–liver axis (Wang et al., 2020; Kotlyarov, 2024), and the gut–spinal circulation mechanism. Modifications in the prevalence of specific HGM bacteria may influence the progression of migraines via the gut–brain axis. For example, the co-colonization of *Clostridium* species alongside other bacterial taxa may contribute to the maintenance of gut homeostasis and inhibit the transition from intestinal disorders to migraines (Sgro et al., 2023). Beneficial HGMs can reduce the frequency and severity of migraine attacks through their anti-inflammatory properties and regulation of neurotransmitters. One study indicated that the modulation of the gut-brain axis by *Faecalibacterium* prausnitzii may alleviate migraine symptoms by reducing neuroinflammation and restoring gut homeostasis (Ardizzone et al., 2024; Zhou et al., 2024). Alterations in *Bifidobacterium* adolescentis could potentially affect migraine symptoms by modulating mechanisms involving inflammatory mediators, neuropeptides, and serotonin pathways (Zhou et al., 2024). Certain gut microbiota can produce short-chain fatty acids, which possess anti-inflammatory properties that may mitigate the progression of migraines. Short-chain fatty acids serve as crucial mediators within the gut–liver–brain axis, impacting both brain function and behavior. They exert systemic effects by engaging in signaling through various pathways, notably G protein-coupled receptors, which play a vital role in facilitating communication between the gut and the brain (Li et al., 2025).


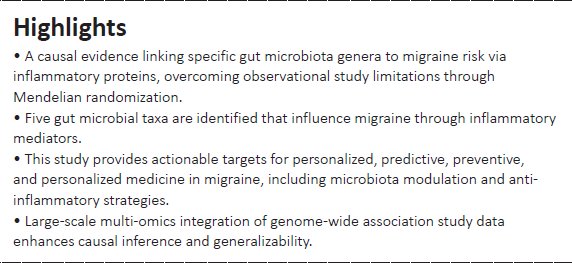


Beyond migraine pathogenesis, the gut microbiota plays a pivotal role in maintaining the balance between neuroinflammation and neural repair processes. Dysbiosis can trigger systemic inflammatory responses that impair the regenerative capacity of the nervous system, while beneficial bacteria produce metabolites such as short-chain fatty acids with anti-inflammatory and neuroprotective properties (Li et al., 2025). Understanding how gut microbiota modulates inflammatory proteins (IPs) may provide insights not only for migraine prevention but also for promoting neural system stability and regeneration.

Migraine is a multifaceted neurological condition that has been linked more frequently to inflammation. Increased levels of cytokines such as tumor necrosis factor-alpha (TNF-α), interleukin-1β, and interleukin-6, along with C-reactive protein and transforming growth factor-β1, have been shown to affect migraine, indicating their roles in neuroinflammation and the development of migraine episodes (Yamanaka et al., 2023). Tang et al. (2020) investigated the role of HGM dysbiosis in exacerbating migraine-like pain through the upregulation of TNF-α. They used broad-spectrum antibiotics and germ-free mice to illustrate that alterations in the HGM can extend the duration of migraine-like pain, indicating that preserving a balanced HGM may be essential for the effective management of migraine symptoms. The complex relationship between HGM and IPs holds substantial implications for elucidating disease mechanisms and formulating therapeutic strategies. Nevertheless, the exact nature of the interactions and the potential combined or synergistic effects of HGM and IPs in the context of migraine remain inadequately understood. Although there is a correlation between intestinal microflora, IPs, and migraine, key gaps still exist. First, existing research is observational, making it impossible to establish causality. Second, there is a lack of direct evidence linking specific intestinal bacterial groups with migraine through specific inflammatory mediators. Third, the degree of regulation—the proportion of the influence of the microbial community on migraine exerted through IPs—is still impossible to quantify. Addressing these gaps is essential for developing targeted microbiome-based interventions and advancing personalized predictive, preventive, and personalized medicine (PPPM) strategies for migraine management.

Mendelian randomization (MR) is a robust methodological approach in genetic research that utilizes Mendel’s laws of inheritance to deduce causal relationships between modifiable exposures and health outcomes while accounting for confounding factors (Thanassoulis, 2013). This approach uses genetic variants as instrumental variables (IVs), enabling the derivation of unconfounded estimates of causal effects. Often referred to as “nature’s randomized trial,” MR effectively mitigates biases commonly associated with observational studies, thereby yielding more reliable evidence (Thanassoulis, 2013). The utility of MR is further enhanced by the growing availability of large-scale genome-wide association studies (GWAS), which have identified numerous genetic variants linked to complex traits. The integration of GWAS with expression quantitative trait loci (eQTL) data facilitates the elucidation of the genetic determinants underlying these complex traits (Porcu et al., 2019).

In conclusion, MR provides a robust methodological framework for investigating the role of IPs in the association between HGM and migraines. Research into PPPM in the context of migraine has increasingly focused on the role of HGM and IPs. Considering this background, our hypothesis posits that dysbiosis of HGM may be more frequently associated with the onset of migraines, with IPs serving as potential mediators. To investigate this hypothesis, we conducted a two-sample MR analysis and a mediating MR analysis to evaluate the association between HGM and migraine, as well as to explore the mediating effect of IPs. Our objective was to identify specific HGM species that may influence the risk of migraine and to assess the potential mediating role of IPs in the pathway linking HGM dysbiosis to the development of migraine.

## Methods

### Study design

This is a two-sample MR study with mediation analysis.

**Figure 1:**
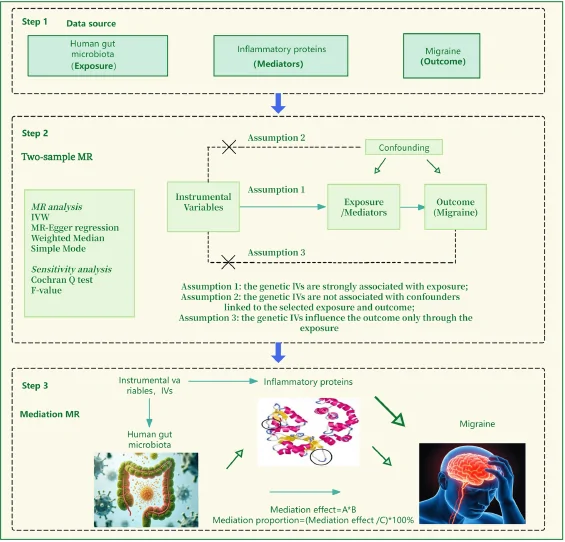
Flowchart of the study. IVs: Instrumental variables; IVW: inverse variance-weighted; MR: Mendelian randomization.

The study flowchart is presented in **Figure 1**. Initially, published summary data from GWAS (https://www.ebi.ac.uk/gwas/) were obtained, which included data on the HGMs, circulating IPs, and migraine (**Additional Table 1**). Subsequently, two-sample MR analyses were performed to assess the causal relationships between HGMs, IPs, and migraine. Finally, two-step analyses were performed to ascertain the mediating role of IPs in the relationship between HGMs and migraine.

**Additional Table 1:** GWAS data sources summary

Data type	Study	PubMed ID	Sample size	Age (yr)	Sex (% female)	GWAS Accession	Data URL
Gut Microbiota	Lopera-Maya 2022	35115690	7,738	18-90 (median 61)	52.30%	GCST90027446-857	https://www.ebi.ac.uk/gwas/
Inflammatory Proteins	Ahola-Olli 2017	27989323	3,200-8,293	24-92 (mean 52±16)	~55%	GCST004420-460	https://www.ebi.ac.uk/gwas/
Inflammatory Proteins	Folkersen 2020	33067605	21,758	24-92 (mean 52±16)	~55%	GCST90012019-109	https://www.ebi.ac.uk/gwas/
Migraine	FinnGen R12	N/A	20,895 cases / 449,141 controls	18-100+ (median 63)	Cases: 74% / Controls: 51%	G6_MIGRAINE	https://www.finngen.fi/

#### Definition of exposures and outcomes

Exposures: (1) Human intestinal microflora: We studied 473 microbial characteristics across multiple taxonomic levels, including 8 phyla, 13 classes, 19 orders, 28 families, 150 genera, and 255 species. The analyses primarily focused on the levels of genus and species, as these provide operational insights for mechanism research. Microbial abundances were center log-ratio (CLR) transformed. (2) Circulating IPs: We examined 91 types of inflammation-related proteins measured by the Olink Target 96 Inflammation panel, expressing them as standardized protein expression units on a log2 scale.

Outcomes: The binary outcome is defined as any recorded diagnosis of migraine (ICD-10: G43) in Finnish national health registries, encompassing both migraine with aura and migraine without aura.

### Data sources

The FINRISK cohort (Ruotsalainen et al., 2021) comprised 5959 Finnish individuals of European ancestry, recruited between 1992 and 2012. The gut microbiota data were sourced from the Dutch Microbiome Project (DMP), which included 7738 Dutch individuals and analyzed 473 taxa across multiple levels, with abundances CLR-transformed. The IPs dataset included 91 proteins from 11 cohorts, totaling 14,824 individuals, with the largest cohort being FINRISK (5959 Finnish individuals). The migraine data were obtained from FinnGen R12, which included 20,895 cases and 449,141 controls of Finnish ancestry.

All data are publicly available from the GWAS Catalog. Participants were genotyped using Illumina whole-genome single nucleotide polymorphism (SNP) arrays (HumanCoreExome, OmniExpress, and 610K platforms). Stringent quality control procedures included: (1) SNP-level filters (minor allele frequency > 1%, genotyping call rate > 95%, Hardy-Weinberg equilibrium *P* > 1 × 10^–6^); (2) sample-level filters (call rate > 95%, excessive heterozygosity |F| > 0.2, cryptic relatedness pi-hat > 0.125); and (3) exclusion of outlier phenotypes (height or body mass index > 5 standard deviations from the mean). Imputation was performed using the Haplotype Reference Consortium panel with IMPUTE2, retaining variants with an imputation quality INFO score > 0.8. Population stratification was controlled using the first 10 genetic principal components derived from linkage disequilibrium (LD)-pruned SNPs. After quality control, 5,967,866 SNPs from 5959 individuals were available for analysis. The genomic inflation factor *λ* = 1.02, indicating adequate control of population structure.

The Olink Target Inflammation panel was used to measure GWAS data for 91 circulating IPs in 11 cohorts, encompassing a total of 14,824 participants of European descent. Publicly available data from FinnGen R12 (https://www.finngen.fi; phenotypeG6_MIGRAINE) were used, which included 20,895 cases and 449,141 controls of Finnish ancestry.

Population characteristics: age ranged from 18 to over 100 years (median age 63 years); among cases, 74% were female and 26% were male; among controls, 51% were female and 49% were male. Migraine cases were defined using ICD-10 code G43 and subcodes (G43.0–G43.9), which included migraine with and without aura, status migrainosus, and other migraine subtypes. The complete ICD-10 code list, along with case numbers and percentages, is provided in **Additional Table 2**. Controls were individuals without any recorded migraine diagnosis. Cases were identified from Finnish national health registries (1969–2021) with diagnostic validation (positive predictive value > 85%). Genotyping was performed using Illumina and Affymetrix arrays; after quality control (call rate > 95%, minor allele frequency > 0.1%, INFO score > 0.6), 20,175,454 variants were available for analysis. The genomic inflation factor *λ* = 1.08 (*λ*_1000_ = 1.02). Additional sample characteristics and data sources are listed in **Additional Tables 1** and **2**.

**Additional Table 2:** ICD-10 diagnostic codes for migraine (FinnGen R12)

ICD-10 Code	Description	Number of Cases	Percentage
G43	Migraine (all subtypes)	20895	1
G43.0	Migraine without aura	8426	0.403
G43.1	Migraine with aura	4127	0.198
G43.2	Status migrainosus	389	0.019
G43.3	Complicated migraine	1052	0.050
G43.8	Other migraine	2518	0.121
G43.9	Migraine, unspecified	4383	0.210

Cases identified from Finnish national health registries (1969-2021). Diagnostic validation: positive predictive value >85%.

### Instrumental variables

IVs were used to investigate the causal relationship between HGMs, IPs, and migraine. MR must meet three criteria: correlation with exposure factors, independence from confounders, and mediation of outcomes solely through exposure factors without a direct association. Initially, SNPs that were significantly associated with the exposures were retained. For each exposure phenotype, SNPs with a significance threshold of *P* < 5 × 10^–6^ were selected. This threshold is less stringent than genome-wide significance (*P* < 5 × 10^–8^) but is widely accepted in MR studies of complex traits, particularly for microbiota and proteins, where genetic associations tend to be modest. This approach ensures a sufficient number of IVs while maintaining instrument strength (all F-statistics > 10). The validity of our findings is supported by consistency across multiple sensitivity analyses.

IV selection: *P* < 5 × 10^–6^ threshold (pragmatic for microbiome/proteome GWAS; Sanna et al., 2019; Kurilshikov et al., 2021). All instruments verified F > 10 via F = *R*^2^(N–K–1)/K(1–*R*^2^). LD-clumping: *r*^2^ < 0.001, 10,000 kb window (1000 Genomes European reference). LD calculations were performed using the 1000 Genomes Project Phase 3 European population reference panel (EUR, *n* = 503) as the LD reference, which is appropriate given that all GWAS datasets were derived from European ancestry populations. This stringent LD threshold ensures that our IVs are independent and reduces bias from correlated variants. In the initial GWAS quality control program, all the instruments SNPs were confirmed to have passed the Hardy-Weinberg balance test (*P* > 1 × 10^–6^). In the process of data generation, snapshots that deviate significantly from HWE are excluded to ensure that our tool variables come from high-quality and genetically reliable variation. This reduces the risk of genotyping errors or population substructure bias in our MR analysis. Importantly, both the effect of single-nucleotide polymorphisms on the exposure and the effect of the exposure on the outcome were attributed to the same allele. During the coordination process, uncertain SNPs with non-consistent alleles and palindromic SNPs with uncertain strands were discarded. Additionally, the strength of each IV was evaluated by calculating the F-statistic for each IV. In our study, the F-statistics were far greater than 10, indicating that these SNPs were strong IVs. To minimize horizontal pleiotropy from potential confounders, instrumental SNPs were queried for associations with body mass index, smoking, and alcohol consumption using PhenoScanner V2 (*P* < 5 × 10^–8^). Sensitivity analyses excluding potentially confounding SNPs did not materially alter the results. Furthermore, MR-PRESSO was performed to detect and correct for horizontal pleiotropic outliers. The global test assessed overall heterogeneity, and outlier-corrected estimates were used when significant heterogeneity was detected (*P* < 0.05). All IV selection and quality control steps were performed using the R package TwoSampleMR (v0.5.8), which is implemented as part of the MR-Base platform. The package is freely available from the MR-Base website (https://www.mrbase.org) and its GitHub repository (https://github.com/MRCIEU/TwoSampleMR).

### Mendelian randomization

The causal relationship between HGMs, IPs, and migraine was assessed using the MR approach. In accordance with standard MR practice, we assumed an additive genetic model for all IVs, wherein each additional effect allele contributes additively to the exposure phenotype. This assumption is consistent with most complex trait genetics and allows for linear modeling of gene-exposure relationships. The primary analysis of our study utilized an inverse variance-weighted (IVW). Additionally, we conducted supplementary analyses via the MR Egger, Weighted median, Simple mode, and Weighted mode. The heterogeneities were quantified using Cochran’s *Q* test, with a *P*-value of less than 0.05 considered indicative of significant heterogeneity. To assess the potential pleiotropic effects of IVs, the MR-Egger regression was used. The directional horizontal Pleiotropy in the causal estimates may be indicated by the intercept term in MR-Egger regression.

Primary MR used IVW method. Sensitivity analyses: MR-Egger, weighted median, mode-based, MR-PRESSO. Diagnostics: F-statistic, Cochran’s Q, MR-Egger intercept, leave-one-out (details in **Additional Tables 3–5**).

Two-step mediation: βa (HGM→IP), βb (IP→migraine), βc (total HGM→migraine). Indirect effect: βind=βa×βb; Direct: βdir = βc-βind; PM = βind/βc. Key points: (1) Three-way sample independence (DMP/protein cohorts/FinnGen); (2) All calculations on log-OR scale; (3) For binary outcomes, PM interpreted as approximate relative contribution, not exact percentage (OR non-collapsibility; Vanderweele and Vansteelandt, 2010); (4) 95% CIs via bootstrap (1000 iterations).

### Assessment of sample overlap

Sample overlap: HGM to Migraine: DMP(Dutch) *vs* FinnGen(Finnish) = no overlap. IP→Migraine: FINRISK(5959) *vs* FinnGen(470,036) = potential < 1.3% overlap (negligible per Burgess et al., 2016). HGM to IP: minimal overlap. For intestinal microflora (FIN risk, *n* = 5959) and migraine (Finn gen R12), both studies included Finnish participants, which increased the possibility of overlapping samples. According to the recruitment period and geographical distribution, we estimated that the potential overlap could affect as much as 10%–20% of the FIN risk sampling. However, the F-statistics of our instrument variables were significantly greater than 10 (median *f* = 28.3), which indicates that strong instruments are not easily influenced by weak instrument bias with overlapping samples. The consistency of findings across multiple sensitivity analyses suggests results were not primarily driven by overlap-induced bias. For IPs (multi-cohort GWAS, *n* = 14,824) and migraine (gen, Finland), GWAS proteins include 11 cohorts from many European countries, and Finland has limited representation, indicating that the sample overlap is the smallest.

### Ethics statement

This study utilized publicly available GWAS summary statistics without individual-level data. All initial GWAS studies have obtained appropriate ethical approvals from the review committees of their respective institutions and informed consent of the participants. Our secondary analysis of summary-level data did not require additional ethical approval.

### Statistical analysis

All analyses were conducted in R (version 4.3.2; R Foundation for Statistical Computing, https://www.r-project.org). MR was performed using the TwoSampleMR package (version 0.5.8; https://github.com/MRCIEU/TwoSampleMR) and pleiotropy assessment with MR-PRESSO (version 1.0; https://github.com/rondolab/MR-PRESSO). SNPs associated with exposures at *P* < 5 × 10^–6^ were selected as IVs to ensure adequate strength for complex traits. LD clumping (*r*^2^ < 0.001, 10,000 kb window) ensured independence, and ambiguous or palindromic SNPs were removed during harmonization. Instrument strength was evaluated using F-statistics (> 10 considered strong). Causal estimates were calculated using IVW, with MR-Egger, weighted median, and mode-based methods as sensitivity analyses. Heterogeneity and pleiotropy were assessed using Cochran’s Q and MR-Egger intercept. Two-step MR mediation was applied to estimate indirect effects. Statistical significance was defined as *P* < 0.05.

## Results

After quality control and IV selection, the final analytical samples comprised: (1) 5259 SNPs as IVs for 473 gut microbiota taxa from 7738 individuals (Dutch Microbiome Project, Lopera-Maya et al., 2021); (2) 1284 SNPs for 91 IPs from 14,824 individuals (11 cohorts); (3) migraine GWAS data with 20,895 cases and 449,141 controls from FinnGen R12. All IVs demonstrated strong instrument strength (F-statistics > 10), indicating minimal weak instrument bias. Baseline characteristics of all GWAS summary data are presented in **Additional Table 3**.

**Additional Table 3:** Baseline characteristics of GWAS summary data

Data source	Study	Total sample	Cases	Controls	Age (yr)	Sex (% female)	Ancestry	GWAS Accession
Gut Microbiota	Lopera-Maya et al. 2022	7,738	7,738 participants	N/A	18-90 (median 61)	52.30%	European (Dutch)	GCST90027446-GCST90027857
Inflammatory Proteins	Ahola-Olli et al. 2017	3,200-8,293*	Varies by protein	N/A	24-92 (mean 52±16)	~55%	Finnish	GCST004420-GCST004460
Inflammatory Proteins	Folkersen et al. 2020	21,758	21,758 participants	N/A	24-92 (mean 52±16)	~55%	European (multi-cohort)	GCST90012019-GCST90012109
Migraine	FinnGen R12	470,036	20,895	449,141	18-100+ (median 63)	Cases: 74%; Controls: 51%	Finnish	Phenotype: G6_MIGRAINE

### Associations of gut microbiota with migraine

**Figure 2:**
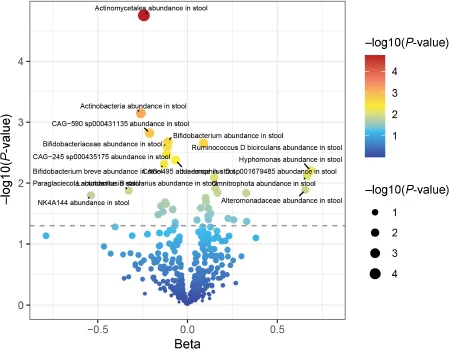
Mendelian randomization estimates for the association between human gut microbiota (including 473 genera) and migraine.

**Figure 3:**
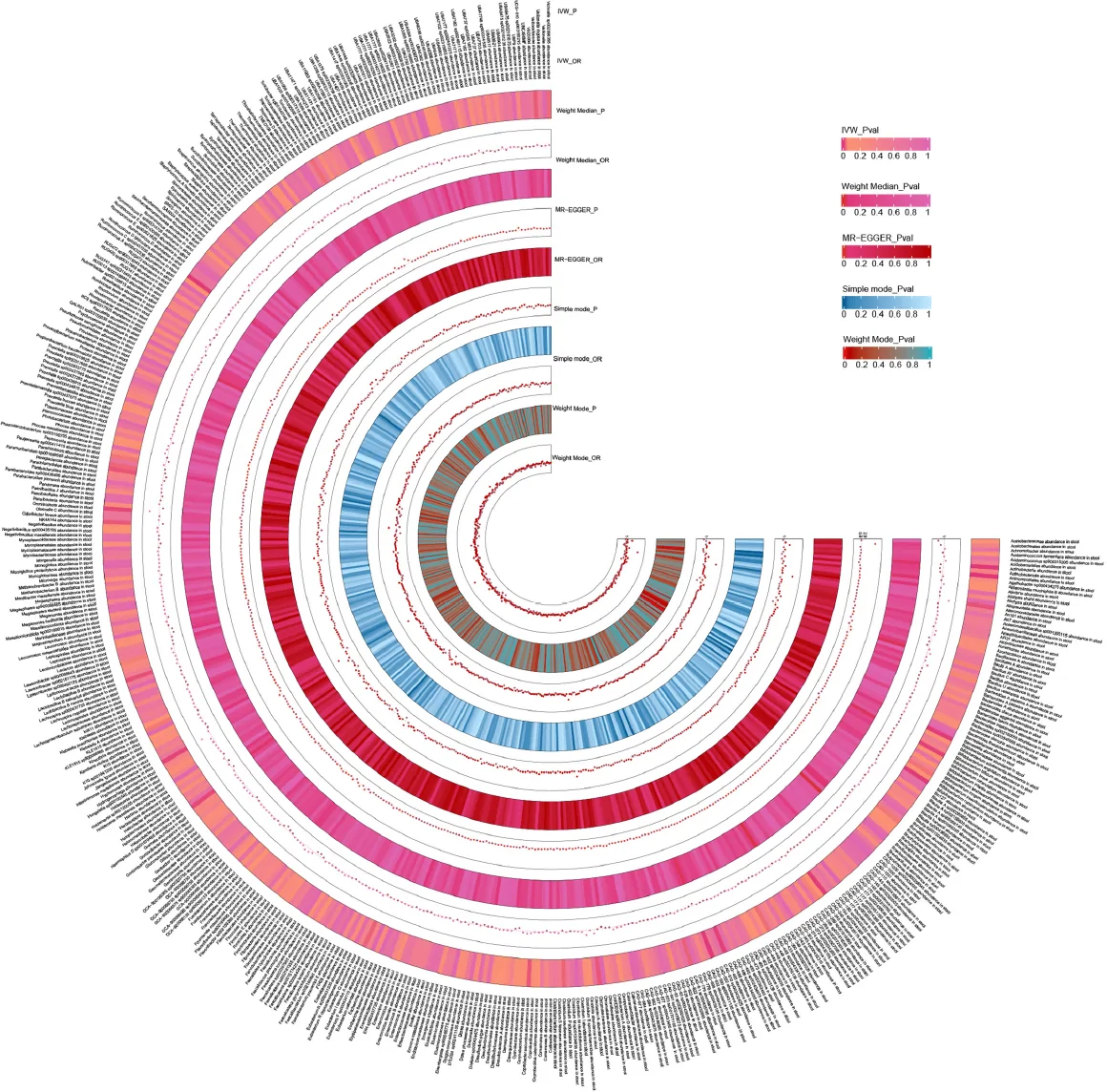
Circular heatmap between human gut microbiota and migraine. Single nucleotide polymorphism influences the causal effect through five Mendelian randomization methods. Each region corresponds to a different level of gut microbiota.

Instrument strength: Median F = 31.7 (range 10.2–156.3); all 473 taxa F > 10. nSNPs: 3–87 (median 15). Details in **Figure 2** and **Additional Table 3**. Thirty-seven gut microbial taxa (2 phyla, 3 families, 28 genera, and 4 species) associated with migraine were identified. Examples include *Actinobacteria* (phylum, OR = 0.772, *P* = 0.003) and *Brachyspiraceae* (family, OR = 0.850, *P* = 0.022). Among these taxa, 18 were found to be protective, while 19 were associated with an increased risk of migraine. Diagnostics revealed that 86.6% of the taxa showed no heterogeneity (Q *P* > 0.05), and no directional pleiotropy was detected (Egger *P* > 0.05 for all 37 taxa). MR-PRESSO identified outliers in 10.2% of cases, while 89.2% of the 37 significant associations had no outliers. Among the identified taxa, 18 genera exhibited protective effects (OR < 1, *P* < 0.05), whereas 19 genera demonstrated positive associations with migraine risk (OR > 1, *P* < 0.05). Notably, five genera played significant roles in subsequent mediation analyses and warrant particular attention: *Actinobacteria* (OR = 0.772, 95% CI = 0.671–0.888, *P* = 2.8 × 10^–4^), *Brachyspiraceae* (OR = 0.850, 95% CI = 0.739–0.977, *P* = 0.022), *CAG-269* sp001915995 (OR = 1.122, 95% CI = 1.010–1.247, *P* = 0.032), *Lawsonibacter* sp002161175 (OR = 1.252, 95% CI = 1.069–1.467, *P* = 0.005), and *Paraglaciecola* (OR = 0.720, 95% CI = 0.562–0.923, *P* = 0.009). The complete list of all 37 genera, along with detailed effect estimates, heterogeneity tests, and pleiotropy assessments, is provided in **Additional Table 4**. **Figure 3** presents a circular heatmap summarizing the causal effects of gut microbiota on migraine estimated by five Mendelian randomization methods.

Sensitivity and heterogeneity analyses: To assess the robustness of our findings, we compared causal estimates across multiple MR methods, including IVW, MR-Egger, weighted median, simple mode, and weighted mode. For the 37 bacterial taxa significantly associated with migraine in the IVW analysis, we observed generally consistent effect directions across different methods, strengthening confidence in our causal inferences.

Heterogeneity was assessed using MR-PRESSO global tests. For the majority of gut microbiota-migraine associations (406/469, 86.6%), no significant heterogeneity was detected (*P* > 0.05), indicating homogeneous causal effects across IVs. Among the 37 significant associations, most demonstrated acceptable heterogeneity levels. For associations with significant heterogeneity (62 genera, *P* < 0.05), MR-PRESSO outlier correction was applied to obtain robust causal estimates. The MR-Egger intercept test did not indicate directional pleiotropy for the majority of associations (*P* > 0.05), supporting the validity of our IVs. Detailed results for all MR methods and heterogeneity tests are provided in **Additional Table 4**.

### Associations of circulating inflammatory proteins with migraine

**Figure 4:**
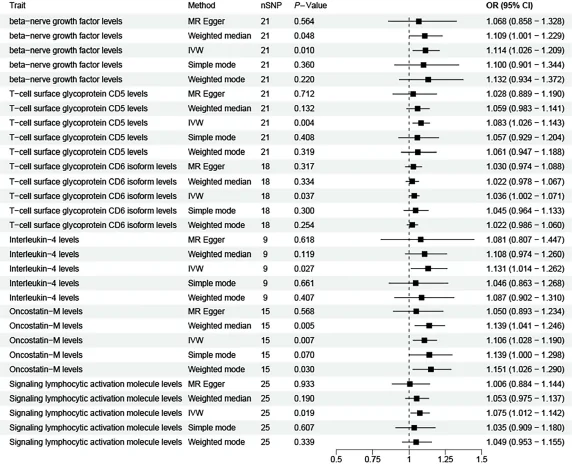
Forest plot visualizing the causal effects of inflammatory proteins on migraine. CI: Confidence interval; IVW: inverse variance-weighted; MR: Mendelian randomization; nSNP: the count of single nucleotide polymorphisms; OR: odds ratio.

A total of 91 IPs were used to perform MR analysis related to migraine. The results indicated that 8 IPs may increase the risk of migraine, including beta-nerve growth factor (β-NGF; IVW: OR = 1.114, 95% CI = 1.026–1.209, *P* = 0.01), T-cell surface glycoprotein CD5 (IVW: OR = 1.083, 95% CI = 1.026–1.143, *P* = 0.004), T-cell surface glycoprotein CD6 isoform (IVW: OR = 1.036, 95% CI = 1.002–1.071, *P* = 0.037), C–X–C motif chemokine 10 (IVW: OR = 1.057, 95% CI = 1.000–1.118, *P* = 0.048), C–X–C motif chemokine 11 (IVW: OR = 1.079, 95% CI = 1.014–1.149, *P* = 0.017), IL-4 (IVW: OR = 1.131, 95% CI = 1.014–1.262, *P* = 0.027), Oncostatin-M (IVW: OR = 1.106, 95% CI = 1.028–1.190, *P* = 0.007), and an unspecified factor (IVW: OR = 1.075, 95% CI = 1.012–1.42, *P* = 0.019). Neither test indicated the presence of potential horizontal pleiotropy or heterogeneity (*P* > 0.05) (**Figure 4** and **Additional Table 4**).

Sensitivity and heterogeneity analyses: Consistency across MR methods was observed for inflammatory protein associated with migraine. All eight proteins showing significant associations in the IVW analysis demonstrated concordant effect directions across sensitivity analyses (MR-Egger, weighted median, and mode-based methods).

Heterogeneity was assessed using MR-PRESSO global tests. Among the 91 proteins, 72 (79.1%) exhibited no significant heterogeneity (*P* > 0.05), suggesting consistent causal effects across instrumental variants. For the 19 proteins (20.9%) showing significant heterogeneity (*P* < 0.05), outlier-corrected estimates were utilized. MR-Egger intercept tests yielded *P* > 0.05 for these associations, indicating the absence of directional pleiotropy.

Importantly, all four IPs involved in the mediation pathways (**Table 1**) showed no significant heterogeneity: β-NGF (*P* = 0.145), IL-4 (*P* = 0.197), Oncostatin-M (*P* = 0.224), and SLAM (*P* = 0.135). This supports the reliability of our mediation analyses and strengthens the validity of the identified causal pathways. Detailed results are provided in **Figure 4** and **Additional Table 4**.

**Table 1:** Mediator analysis results of human gut microbiota, circulating inflammatory proteins, and migraine risk

Human gut microbiota	Inflammatory proteins	Path a βa	Path b βb	Total effect βc	Direct effect β’c	Indirect effect βa*b	Proportion mediated (%)	PM_95% CI_lower	PM_95% CI_upper	Biological mechanism
*Actinobacteria* abundance in stool	Beta-nerve growth factor level	–0.289	0.108	-0.259	-0.229	-0.0312	0.12	–0.176	0.417	*Actinobacteria* modulates neurotrophin signaling; beta-nerve growth factor promotes nociceptor sensitization.
*Brachyspiraceae* abundance in stool	IL-4 level	–0.252	0.123	–0.163	–0.133	–0.031	0.19	–0.905	1.285	*Brachyspiraceae* influences immune homeostasis; IL-4 shifts macrophage polarization.
*CAG-269* sp001915995 abundance in stool	IL-4 level	0.146	0.123	0.116	0.098	0.018	0.155	–1.14	1.452	*CAG-269* alters gut immune responses; IL-4 modulates neuroinflammation.
*Lawsonibacter* sp002161175 abundance in stool	Oncostatin-M level	0.349	0.1	0.225	0.19	0.035	0.156	–0.525	0.838	*Lawsonibacter* affects cytokine production; Oncostatin-M induces IL-6 family-mediated neuroinflammation.
*Lawsonibacter* sp002161175 abundance in stool	Signaling lymphocytic activation molecule level	0.356	0.072	0.225	0.2	0.025	0.113	–0.381	0.609	*Lawsonibacter* modulates immune signaling; signaling lymphocytic activation molecule regulates T-cell activation.
*Paraglaciecola* abundance in stool	Signaling lymphocytic activation molecule levels	–0.308	0.072	–0.327	–0.306	–0.022	0.067	–0.117	0.252	*Paraglaciecola* influences immune function; signaling lymphocytic activation molecule co-stimulates inflammatory pathways.

IL: Interleukin.

### Association between human gut microbiota and circulating inflammatory proteins

**Figure 5:**
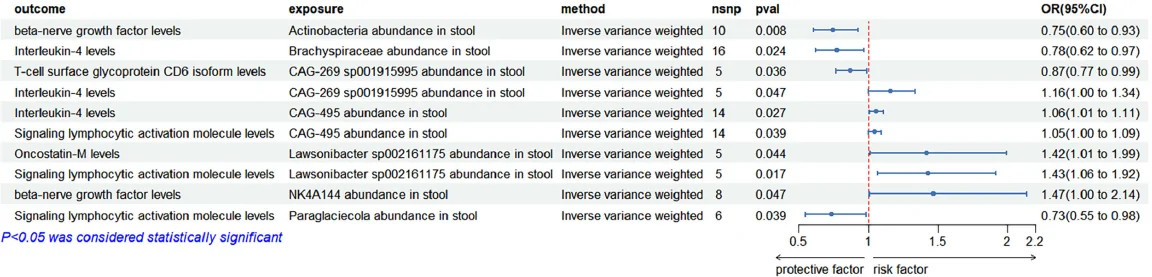
Forest plot of human gut microbiota and circulating inflammatory proteins. CI: Confidence interval; nSNP: the count of single nucleotide polymorphisms; OR: odds ratio.

Among the associations between gut microbiota and IPs (**Figure 5** and **Additional Table 5**), ten significant associations were identified. Four genera were found to reduce inflammatory protein levels: *Actinobacteria* decreased β-NGF (OR = 0.749, 95% CI = 0.604–0.928, *P* = 0.008), *Brachyspiraceae* reduced IL-4 (OR = 0.777, 95% CI = 0.624–0.968, *P* = 0.024), *CAG-269* sp001915995 lowered CD6 (OR = 0.872, 95% CI = 0.768–0.991, *P* = 0.036), and *Paraglaciecola* decreased SLAM (OR = 0.735, 95% CI = 0.548–0.985, *P* = 0.039). Conversely, six associations were linked to increased inflammatory protein production: CAG-269 sp001915995 increased IL-4 (OR = 1.158, 95% CI = 1.002–1.338, *P* = 0.047), CAG-495 elevated IL-4 (OR = 1.056, 95% CI = 1.006–1.108, *P* = 0.027) and SLAM (OR = 1.047, 95% CI = 1.002–1.094, *P* = 0.039), Lawsonibacter sp002161175 increased Oncostatin-M (OR = 1.419, 95% CI = 1.010–1.994, *P* = 0.044) and SLAM (OR = 1.429, 95% CI = 1.065–1.917, *P* = 0.017), and NK4A144 increased β-NGF (OR = 1.466, 95% CI = 1.005–2.138, *P* = 0.047).

Sensitivity and heterogeneity analyses: MR-PRESSO global tests were conducted for 296 gut microbiota-inflammatory protein associations. The majority (254 associations, 85.8%) showed no significant heterogeneity (*P* > 0.05), while 42 associations (14.2%) exhibited heterogeneity (*P* < 0.05). Outlier-corrected estimates were utilized for associations with significant heterogeneity. Notably, all 10 associations involved in the mediation pathways demonstrated acceptable levels of heterogeneity, supporting the robustness of our findings. MR-Egger intercept tests indicated no directional pleiotropy (*P* > 0.05) for any of the associations. Detailed results are provided in **Additional Table 6**.

### Mediation effect of human gut microbiota on migraine outcomes via inflammatory proteins

**Figure 6:**
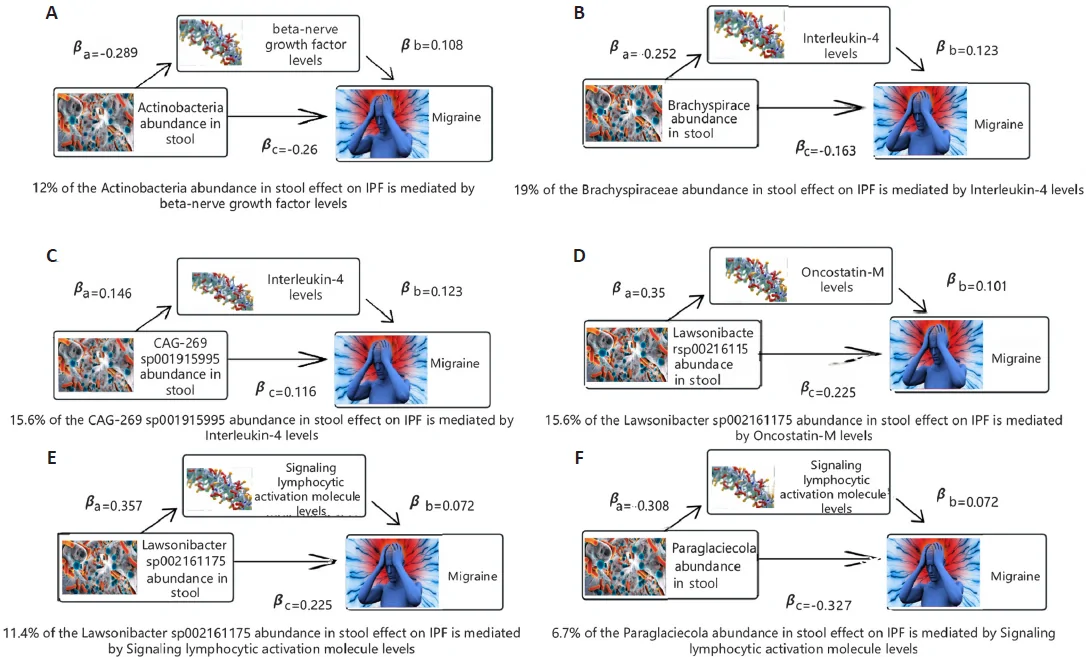
Proportion of gut microbiota in migraine via circulating inflammatory proteins (A–F).

To investigate whether IPs mediate the effects of gut microbiota on migraine, we conducted a mediation analysis using effect estimates from two-step MR. This analysis identified six significant mediation pathways involving five gut microbiota taxa that showed associations in both HGM→IP and IP→migraine analyses (**Table 1** and **Figure 6**). The proportion mediated ranged from 6.7% to 19.0%: *Brachyspiraceae* (family) → IL-4 → migraine (19.0%, 95% CI: –90.5%, 128.5%); *CAG-269* sp001915995 (species) → IL-4 → migraine (15.5%, 95% CI: –114.0%, 145.2%); *Lawsonibacter* sp002161175 (species) → Oncostatin-M → migraine (15.6%, 95% CI: –52.5%, 83.8%); *Actinobacteria* (phylum) → β-NGF → migraine (12.0%, 95% CI: –17.6%, 41.7%); *Lawsonibacter* sp002161175 (species) → SLAM → migraine (11.3%, 95% CI: –38.1%, 60.9%); and *Paraglaciecola* (genus) → SLAM → migraine (6.7%, 95% CI: –11.7%, 25.2%). The proportion mediated represents the approximate percentage of the total effect of each gut microbiota on migraine operating through the specified inflammatory protein pathway, calculated on the log odds ratio scale using the product method, with 95% CIs obtained via bootstrap resampling (1000 iterations).

For binary outcomes, these estimates should be interpreted as relative measures of pathway importance rather than exact percentages due to the non-collapsibility of odds ratios (Vanderweele and Vansteelandt, 2010). The wide confidence intervals (e.g., *Brachyspiraceae*-IL-4: –90.5% to 128.5%) reflect substantial uncertainty inherent in two-step MR mediation analyses due to error propagation across multiple stages, which is common and expected. The modest mediation proportions (all < 20%) suggest that IPs explain only part of the microbiota effects, with remaining effects likely operating through other mediators such as bacterial metabolites, neurotransmitter precursors, or gut barrier integrity. Among the identified taxa, *Paraglaciecola* (typically marine) requires validation. Possible explanations for its detection include dietary exposure, taxonomic misannotation, or closely related human-adapted species. We recommend cautious interpretation of this pathway pending confirmation via quantitative polymerase chain reaction or alternative profiling methods. The other four taxa (*Actinobacteria*, *Brachyspiraceae*, *CAG-269*, *Lawsonibacter*) are well-documented gut commensals with established immune and inflammatory roles, supporting the overall findings.

## Discussion

In this study, we used MR and intermediary analysis to investigate the causal relationships between intestinal microflora, IPs, and migraine. Five specific intestinal microflora affect the risk of migraine through the IP pathway, with mediation ratios ranging from 6.7% to 19%. These findings provide genetic evidence for the gut-brain axis hypothesis in the pathogenesis of migraine and identify potential therapeutic targets.

*Actinobacteria* demonstrated protective effects through β-NGF modulation (12% mediation), consistent with previous studies showing neurotrophic factor modulation (Gorenshtein et al., 2025; Mugo et al., 2025). We observed that the decrease in β-NGF has a protective effect, which may seem contradictory; however, this can be explained by the dual functions of NGF. While NGF supports the health of neurons, increased levels can sensitize the pain pathway through TrkA and p75 NTR receptors, leading to migraine-related allodynia and hyperalgesia (García-Domínguez, 2025). *Brachyspiraceae* exhibited the strongest regulatory effect (19%) through the reduction of IL-4. Although IL-4 is traditionally considered anti-inflammatory via M1 to M2 macrophage polarization (Kiguchi et al., 2015) and opioid peptide release (Labuz et al., 2021), increased levels of IL-4 have been observed in experimental migraine models (Yamanaka et al., 2023; Sun et al., 2024), supporting the pro-inflammatory effects that depend on the situation. *Lawsonibacterium* sp002161175, a butyrate-producing bacterium (Sakamoto et al., 2018), along with Oncostatin-M (15.6%) and SLAM (11.3%), showed pro-inflammatory effects. Oncostatin-M has protective effects on the central nervous system, but it also exhibits pro-inflammatory peripheral effects (Janssens et al., 2015), suggesting that *Lawsonibacter*-induced OSM triggers peripheral inflammatory cascades. The involvement of the SLAM family represents a new discovery, as these co-stimulatory receptors regulate immune responses (Ishibashi et al., 2021), although they have not been previously associated with migraine.

The gut microbiota-migraine relationship operates through multiple pathways. Dietary factors interact with intestinal microflora to regulate neuritis (Gazerani et al., 2024), while symbiosis exacerbates migraine-like pain by upregulating TNF-α (Tang et al., 2020). Disruptions in the gut–liver axis influence migraine through systemic inflammation and altered metabolic pathways (Kotlyarov, 2024), with gut microbiota modulating liver gene expression that affects neurological conditions (Silveira et al., 2022), as well as regulating intestinal peptides, which impact the vagus nerve pathway and metabolism (Wang et al., 2020). The moderate regulation ratio (6.7%–19%) indicates that IPs represent only one component, with other mechanisms including neurotransmitter regulation, metabolite production, and intestinal barrier integrity.

These findings provide mechanistic hypotheses that require extensive validation before clinical application: i. Identification of taxa/proteins as risk biomarkers; ii. Replication in diverse populations, establishment of clinically meaningful cutoffs, and longitudinal validation; iii. Microbiota modulation (through probiotics or diet) or anti-inflammatory strategies may reduce risk; iv. Microbiota profiling could guide treatment, necessitating proof-of-concept studies and identification of treatment response biomarkers; v. Validation priorities should include gnotobiotic models, metabolomics, pilot randomized controlled trials, and prospective cohorts. While MR controls for genetic confounding, interventions may face confounding factors such as diet (triggers, fiber), medications (non-steroidal anti-inflammatory drugs, triptans alter microbiota/inflammation), comorbidities (irritable bowel syndrome, depression), and lifestyle factors (stress, sleep, exercise). Multi-factorial approaches are needed. vi. Translation of these findings into clinical practice may take 10–15 years. The findings are hypothesis-generating and await interventional trials before clinical adoption. Targeted modulation of *Actinobacteria*, *Brachyspiraceae*, and *Lawsonibacter* through prebiotics, probiotics, or dietary interventions is a preventive strategy. From a regenerative medicine perspective, protective bacteria produce metabolites that support neuroplasticity and tissue homeostasis, while the identified IPs (β-NGF promoting neuron survival, IL-4 providing neuroprotection, and Oncostatin-M supporting oligodendrocyte differentiation) indicate that targeted gut microbiota regulation can promote nervous system stability and regenerative capacity (Zhou et al., 2024).

From the perspective of regenerative medicine, our findings hold significance beyond migraine treatment. The identified intestinal microbial communities and IPs are potential targets for regulating the neuroinflammatory environment that affects nerve repair and regeneration. It is known that *actinomycetes* and *bifidobacteria*, which have protective effects, produce metabolites that support neuroplasticity and tissue homeostasis. The established inflammatory protein β-NGF promotes neuron survival and axon growth (García-Domínguez, 2025), while IL-4 plays a neuroprotective role through anti-inflammatory mechanisms (Labuz et al., 2021). Additionally, Oncogen-m supports oligodendrocyte differentiation and myelin repair. These findings suggest that the targeted regulation of specific intestinal microflora can be utilized as a nutritional and immune regulation strategy to enhance the stability and regenerative capacity of the nervous system, representing a promising approach to integrate interventions based on microflora into regenerative neurology.

### Methodological considerations

Our two-sample MR analysis (IVW, MR-Egger, weighted median, MR-PRESSO) is appropriate given the strong instruments (F > 10), minimal overlap, and consistent findings. Recent advances offer opportunities for future refinement: MAGIC could estimate mediation effects while simultaneously accounting for correlated mediators. RIVW/MRBEE addresses winner’s curse and measurement error (Kang et al., 2016; Zhao et al., 2019), although our strong F-statistics suggest minimal bias. MR-RAPS/CAUSE provides robustness against pleiotropy (Morrison et al., 2020); while our MR-Egger/PRESSO detected no directional pleiotropy, these methods could still offer triangulation. Consistency across multiple methods provides reasonable confidence in our causal inferences.

Several limitations warrant consideration in this study. First, mediation proportion estimates for binary outcomes should be interpreted as approximate relative contributions rather than exact percentages due to the non-collapsibility of odds ratios (Vanderweele and Vansteelandt, 2010). Wide confidence intervals reflect uncertainty in two-step estimation. Second, the compositionality of microbiome data may induce spurious associations despite CLR transformation. Third, some taxa (e.g., *Paraglaciecola*) have questionable biological plausibility and require validation, though most identified taxa are established gut commensals. Fourth, Olink measurements represent circulating rather than tissue-specific inflammatory levels. Fifth, the use of European-only populations limits generalizability. Sixth, although sensitivity analyses detected no directional pleiotropy, subtle effects cannot be excluded. Seventh, some taxa had fewer SNPs (nsnp < 10), limiting the applicability of certain sensitivity methods. Eighth, MR cannot elucidate precise mechanisms, which require experimental validation. Finally, summary-level data precluded subgroup analyses by migraine subtypes, sex, or age.

In conclusion, the study conducted by MR elucidated the relationship between HGMs, IPs, and migraine. These findings offer potential insights into the pathogenesis, diagnosis, and treatment of migraine. Understanding migraine and the specific role of IPs in causal interactions with certain HGMs enables researchers and clinicians to formulate more effective PPPM strategies, which could lead to improved prevention, early detection, and personalized migraine care.

## Additional files:

***Additional Table 1:*** *GWAS data sources summary.*

***Additional Table 2:*** *ICD-10 diagnostic codes for migraine (FinnGen R12).*

***Additional Table 3:*** *Baseline characteristics of GWAS summary data.*

***Additional Table 4:*** *MR-PRESSO results of associations between gut microbiota and migraine.*

Additional Table 4 MR-PRESSO results of associations between gut microbiota and migraine

***Additional Table 5:*** *MR-PRESSO results of associations between inflammatory proteins and migraine.*

Additional Table 5 MR-PRESSO results of associations between inflammatory proteins and migraine

***Additional Table 6:*** *MR-PRESSO results of associations between gut microbiota and inflammatory proteins.*

Additional Table 6 MR-PRESSO results of associations between gut microbiota and inflammatory proteins

## Data Availability

*The data that support the findings of this study are openly available in GWAS database at https://www.ebi.ac.uk/gwas/.*
