# Supplementary material for: Two-sample Mendelian randomization study of gut microbiota and inflammatory proteins: Predictive, preventive, and personalized treatment for migraine
Source: Neural Regen Res. 2026 Feb 5;21(9):4474–80. doi: 10.4103/NRR.NRR-D-25-01711 (PMC13557522; doi:10.4103/NRR.NRR-D-25-01711)
Supplement: Supplementary file 1 [file NRR-21-4474_Suppl1.pdf]

**Additional Table 4 MR-PRESSO results of associations between gut microbiota and migraine**

| FolderName | `MR-PRESSO results`$`Global Test`$Pvalue |
|---|---|
| GCST90032172 | 0.341 |
| GCST90032173 | 0.12 |
| GCST90032174 | 0.047 |
| GCST90032175 | 0.176 |
| GCST90032176 | 0.713 |
| GCST90032177 | 0.738 |
| GCST90032178 | 0.135 |
| GCST90032179 | 0.358 |
| GCST90032181 | 0.8 |
| GCST90032182 | 0.734 |
| GCST90032183 | 0.724 |
| GCST90032184 | 0.658 |
| GCST90032185 | 0.09 |
| GCST90032186 | 0.196 |
| GCST90032187 | 0.632 |
| GCST90032188 | 0.634 |
| GCST90032189 | 0.166 |
| GCST90032190 | 0.557 |
| GCST90032191 | 0.211 |
| GCST90032192 | 0.558 |
| GCST90032193 | 0.758 |
| GCST90032194 | 0.426 |
| GCST90032195 | 0.531 |
| GCST90032196 | 0.076 |
| GCST90032197 | 0.691 |
| GCST90032198 | 0.472 |
| GCST90032199 | 0.308 |
| GCST90032200 | 0.007 |
| GCST90032201 | 0.054 |
| GCST90032202 | 0.72 |
| GCST90032203 | 0.981 |
| GCST90032204 | 0.547 |
| GCST90032205 | 0.107 |
| GCST90032206 | 0.377 |
| GCST90032207 | 0.025 |
| GCST90032208 | 0.019 |
| GCST90032209 | 0.499 |
| GCST90032210 | 0.197 |
| GCST90032211 | 0.872 |
| GCST90032212 | 0.22 |
| GCST90032213 | 0.356 |
| GCST90032214 | 0.213 |
| GCST90032215 | 0.479 |
| GCST90032216 | 0.864 |
| GCST90032217 | 0.845 |
| GCST90032218 | 0.95 |
| GCST90032219 | 0.424 |
| GCST90032220 | 0.078 |
| GCST90032221 | 0.049 |
| GCST90032222 | 0.009 |
| GCST90032223 | 0.316 |

| | |
|---|---|
| GCST90032224 | 0.083 |
| GCST90032225 | 0.093 |
| GCST90032226 | 0.056 |
| GCST90032227 | 0.039 |
| GCST90032228 | 0.261 |
| GCST90032229 | 0.062 |
| GCST90032230 | 0.505 |
| GCST90032231 | 0.831 |
| GCST90032232 | 0.243 |
| GCST90032233 | 0.1 |
| GCST90032234 | 0.595 |
| GCST90032235 | 0.239 |
| GCST90032236 | 0.719 |
| GCST90032237 | 0.177 |
| GCST90032238 | 0.933 |
| GCST90032239 | 0.597 |
| GCST90032240 | 0.502 |
| GCST90032241 | 0.181 |
| GCST90032242 | 0.165 |
| GCST90032243 | 0.191 |
| GCST90032244 | 0.231 |
| GCST90032245 | 0.003 |
| GCST90032246 | 0.009 |
| GCST90032247 | 0.018 |
| GCST90032248 | 0.041 |
| GCST90032249 | 0.403 |
| GCST90032250 | 0.326 |
| GCST90032251 | 0.042 |
| GCST90032252 | 0.045 |
| GCST90032253 | 0.994 |
| GCST90032254 | 0.505 |
| GCST90032255 | 0.753 |
| GCST90032256 | 0.078 |
| GCST90032257 | 0.343 |
| GCST90032258 | 0.169 |
| GCST90032259 | 0.29 |
| GCST90032260 | 0.316 |
| GCST90032261 | 0.335 |
| GCST90032262 | 0.111 |
| GCST90032263 | 0.978 |
| GCST90032264 | 0.351 |
| GCST90032265 | 0.525 |
| GCST90032266 | 0.751 |
| GCST90032267 | 0.576 |
| GCST90032268 | 0.692 |
| GCST90032269 | 0.073 |
| GCST90032270 | 0.277 |
| GCST90032271 | 0.18 |
| GCST90032272 | 0.097 |
| GCST90032273 | 0.669 |
| GCST90032274 | 0.559 |
| GCST90032275 | 0.385 |
| GCST90032276 | 0.545 |
| GCST90032277 | 0.263 |
| GCST90032278 | 0.315 |
| GCST90032279 | 0.877 |
| GCST90032280 | 0.664 |
| GCST90032281 | 0.12 |

| | |
|---|---|
| GCST90032282 | 0.139 |
| GCST90032283 | 0.404 |
| GCST90032284 | 0.6 |
| GCST90032285 | 0.006 |
| GCST90032286 | 0.13 |
| GCST90032287 | 0.892 |
| GCST90032288 | 0.017 |
| GCST90032289 | 0.09 |
| GCST90032290 | 0.16 |
| GCST90032291 | 0.11 |
| GCST90032292 | 0.373 |
| GCST90032293 | 0.252 |
| GCST90032294 | 0.099 |
| GCST90032295 | 0.528 |
| GCST90032296 | 0.968 |
| GCST90032297 | 0.985 |
| GCST90032298 | 0.161 |
| GCST90032299 | 0.02 |
| GCST90032300 | 0.611 |
| GCST90032301 | 0.269 |
| GCST90032302 | 0.937 |
| GCST90032303 | 0.386 |
| GCST90032304 | 0.312 |
| GCST90032305 | 0.923 |
| GCST90032306 | 0.429 |
| GCST90032307 | 0.663 |
| GCST90032308 | 0.053 |
| GCST90032309 | 0.401 |
| GCST90032310 | 0.366 |
| GCST90032311 | 0.211 |
| GCST90032312 | 0.088 |
| GCST90032313 | 0.115 |
| GCST90032314 | 0.895 |
| GCST90032315 | 0.844 |
| GCST90032316 | 0.025 |
| GCST90032317 | 0.244 |
| GCST90032318 | 0.262 |
| GCST90032319 | 0.73 |
| GCST90032320 | 0.539 |
| GCST90032321 | 0.104 |
| GCST90032322 | 0.879 |
| GCST90032323 | 0.884 |
| GCST90032324 | 0.2 |
| GCST90032325 | 0.279 |
| GCST90032326 | 0.865 |
| GCST90032327 | 0.095 |
| GCST90032328 | 0.145 |
| GCST90032329 | 0.445 |
| GCST90032330 | 0.151 |
| GCST90032331 | 0.799 |
| GCST90032332 | 0.597 |
| GCST90032333 | 0.432 |
| GCST90032334 | 0.403 |
| GCST90032335 | 0.603 |
| GCST90032336 | 0.464 |
| GCST90032337 | 0.706 |
| GCST90032338 | 0.24 |
| GCST90032339 | 0.154 |

| | |
|---|---|
| GCST90032340 | 0.367 |
| GCST90032341 | 0.359 |
| GCST90032342 | 0.429 |
| GCST90032343 | 0.341 |
| GCST90032344 | 0.048 |
| GCST90032345 | 0.042 |
| GCST90032346 | 0.004 |
| GCST90032347 | 0.151 |
| GCST90032348 | 0.57 |
| GCST90032349 | 0.677 |
| GCST90032350 | 0.036 |
| GCST90032351 | 0.022 |
| GCST90032352 | 0.003 |
| GCST90032353 | 0.048 |
| GCST90032354 | 0.076 |
| GCST90032355 | 0.954 |
| GCST90032356 | 0.723 |
| GCST90032357 | 0.397 |
| GCST90032358 | 0.257 |
| GCST90032359 | 0.273 |
| GCST90032360 | 0.316 |
| GCST90032361 | 0.056 |
| GCST90032362 | 0.028 |
| GCST90032363 | 0.049 |
| GCST90032364 | 0.714 |
| GCST90032365 | 0.553 |
| GCST90032366 | 0.751 |
| GCST90032367 | 0.673 |
| GCST90032368 | 0.134 |
| GCST90032369 | 0.884 |
| GCST90032370 | 0.448 |
| GCST90032371 | 0.147 |
| GCST90032372 | 0.315 |
| GCST90032373 | 0.978 |
| GCST90032374 | 0.371 |
| GCST90032375 | 0.868 |
| GCST90032376 | 0.402 |
| GCST90032377 | 0.405 |
| GCST90032378 | 0.089 |
| GCST90032379 | 0.341 |
| GCST90032380 | 0.542 |
| GCST90032381 | 0.886 |
| GCST90032382 | 0.504 |
| GCST90032383 | 0.099 |
| GCST90032384 | 0.08 |
| GCST90032385 | 0.612 |
| GCST90032386 | 0.698 |
| GCST90032387 | 0.001 |
| GCST90032388 | 0.212 |
| GCST90032389 | 0.379 |
| GCST90032390 | 0.229 |
| GCST90032391 | 0.041 |
| GCST90032392 | 0.787 |
| GCST90032393 | 0.628 |
| GCST90032394 | 0.138 |
| GCST90032395 | 0.777 |
| GCST90032396 | 0.006 |
| GCST90032397 | 0.245 |

| | |
|---|---|
| GCST90032398 | 0.175 |
| GCST90032399 | 0.029 |
| GCST90032400 | 0.78 |
| GCST90032401 | 0.168 |
| GCST90032402 | 0.7 |
| GCST90032403 | 0.041 |
| GCST90032404 | 0.242 |
| GCST90032405 | 0.164 |
| GCST90032406 | 0.558 |
| GCST90032407 | 0.314 |
| GCST90032408 | 0.863 |
| GCST90032409 | 0.574 |
| GCST90032410 | 0.872 |
| GCST90032411 | 0.668 |
| GCST90032412 | 0.605 |
| GCST90032413 | 0.307 |
| GCST90032414 | 0.59 |
| GCST90032415 | 0.256 |
| GCST90032416 | 0.193 |
| GCST90032417 | 0.89 |
| GCST90032418 | 0.001 |
| GCST90032419 | 0.258 |
| GCST90032420 | 0.249 |
| GCST90032421 | 0.163 |
| GCST90032422 | 0.105 |
| GCST90032423 | 0.368 |
| GCST90032424 | 0.569 |
| GCST90032425 | 0.19 |
| GCST90032426 | 0.001 |
| GCST90032427 | 0.058 |
| GCST90032428 | 0.053 |
| GCST90032429 | 0.228 |
| GCST90032430 | 0.721 |
| GCST90032431 | 0.008 |
| GCST90032432 | 0.072 |
| GCST90032433 | 0.189 |
| GCST90032434 | 0.976 |
| GCST90032435 | 0.541 |
| GCST90032436 | 0.915 |
| GCST90032437 | 0.046 |
| GCST90032438 | 0.185 |
| GCST90032439 | 0.442 |
| GCST90032440 | 0.212 |
| GCST90032441 | 0.469 |
| GCST90032442 | 0.241 |
| GCST90032443 | 0.749 |
| GCST90032444 | 0.576 |
| GCST90032445 | 0.381 |
| GCST90032446 | 0.155 |
| GCST90032447 | 0.821 |
| GCST90032448 | 0.205 |
| GCST90032449 | 0.561 |
| GCST90032450 | 0.001 |
| GCST90032451 | 0.006 |
| GCST90032452 | 0.009 |
| GCST90032453 | 0.016 |
| GCST90032454 | 0.071 |
| GCST90032455 | 0.493 |

| | |
|---|---|
| GCST90032456 | 0.012 |
| GCST90032457 | 0.476 |
| GCST90032458 | 0.288 |
| GCST90032459 | 0.899 |
| GCST90032460 | 0.253 |
| GCST90032461 | 0.166 |
| GCST90032462 | 0.359 |
| GCST90032463 | 0.005 |
| GCST90032464 | 0.036 |
| GCST90032465 | 0.061 |
| GCST90032466 | 0.064 |
| GCST90032467 | 0.222 |
| GCST90032468 | 0.191 |
| GCST90032469 | 0.972 |
| GCST90032470 | 0.54 |
| GCST90032471 | 0.839 |
| GCST90032472 | 0.807 |
| GCST90032473 | 0.643 |
| GCST90032474 | 1 |
| GCST90032475 | 0.734 |
| GCST90032476 | 0.52 |
| GCST90032477 | 0.925 |
| GCST90032478 | 0.594 |
| GCST90032479 | 0.022 |
| GCST90032480 | 0.261 |
| GCST90032481 | 0.07 |
| GCST90032482 | 0.066 |
| GCST90032483 | 0.006 |
| GCST90032484 | 0.974 |
| GCST90032485 | 0.246 |
| GCST90032486 | 0.177 |
| GCST90032487 | 0.141 |
| GCST90032488 | 0.058 |
| GCST90032489 | 0.027 |
| GCST90032490 | 0.09 |
| GCST90032491 | 0.049 |
| GCST90032492 | 0.533 |
| GCST90032493 | 0.649 |
| GCST90032494 | 0.81 |
| GCST90032495 | 0.94 |
| GCST90032496 | 0.98 |
| GCST90032497 | 0.449 |
| GCST90032498 | 0.367 |
| GCST90032499 | 0.096 |
| GCST90032500 | 0.783 |
| GCST90032501 | 0.069 |
| GCST90032502 | 0.178 |
| GCST90032503 | 0.572 |
| GCST90032504 | 0.361 |
| GCST90032505 | 0.405 |
| GCST90032506 | 0.679 |
| GCST90032507 | 0.09 |
| GCST90032508 | 0.866 |
| GCST90032509 | 0.081 |
| GCST90032510 | 0.039 |
| GCST90032511 | 0.705 |
| GCST90032512 | 0.138 |
| GCST90032513 | 0.041 |

| | |
|---|---|
| GCST90032514 | 0.785 |
| GCST90032515 | 0.384 |
| GCST90032516 | 0.177 |
| GCST90032517 | 0.226 |
| GCST90032518 | 0.679 |
| GCST90032519 | 0.449 |
| GCST90032520 | 0.148 |
| GCST90032521 | 0.973 |
| GCST90032522 | 0.253 |
| GCST90032523 | 0.931 |
| GCST90032524 | 0.03 |
| GCST90032525 | 0.281 |
| GCST90032526 | 0.14 |
| GCST90032527 | 0.998 |
| GCST90032528 | 0.955 |
| GCST90032529 | 0.803 |
| GCST90032530 | 0.561 |
| GCST90032531 | 0.538 |
| GCST90032532 | 0.22 |
| GCST90032533 | 0.479 |
| GCST90032534 | 0.594 |
| GCST90032535 | 0.2 |
| GCST90032536 | 0.777 |
| GCST90032537 | 0.615 |
| GCST90032538 | 0.736 |
| GCST90032539 | 0.686 |
| GCST90032541 | 0.991 |
| GCST90032542 | 0.114 |
| GCST90032545 | 0.653 |
| GCST90032546 | 0.264 |
| GCST90032547 | 0.205 |
| GCST90032548 | 0.409 |
| GCST90032549 | 0.131 |
| GCST90032550 | 0.489 |
| GCST90032551 | 0.218 |
| GCST90032552 | 0.147 |
| GCST90032553 | 0.054 |
| GCST90032554 | 0.05 |
| GCST90032555 | 0.302 |
| GCST90032556 | 0.493 |
| GCST90032557 | 0.449 |
| GCST90032558 | 0.126 |
| GCST90032559 | 0.459 |
| GCST90032560 | 0.866 |
| GCST90032561 | 0.56 |
| GCST90032562 | 0.34 |
| GCST90032563 | 0.201 |
| GCST90032564 | "<0.001" |
| GCST90032565 | 0.952 |
| GCST90032566 | 0.883 |
| GCST90032567 | 0.198 |
| GCST90032568 | 0.525 |
| GCST90032569 | 0.187 |
| GCST90032570 | 0.299 |
| GCST90032571 | 0.247 |
| GCST90032572 | 0.815 |
| GCST90032573 | 0.648 |
| GCST90032574 | 0.172 |

| | |
|---|---|
| GCST90032575 | 0.011 |
| GCST90032576 | 0.604 |
| GCST90032577 | 0.113 |
| GCST90032578 | 0.115 |
| GCST90032579 | 0.021 |
| GCST90032580 | 0.019 |
| GCST90032581 | 0.277 |
| GCST90032582 | 0.347 |
| GCST90032583 | 0.62 |
| GCST90032584 | 0.02 |
| GCST90032585 | 0.08 |
| GCST90032586 | 0.253 |
| GCST90032587 | 0.002 |
| GCST90032588 | 0.083 |
| GCST90032589 | 0.002 |
| GCST90032590 | 0.851 |
| GCST90032591 | 0.822 |
| GCST90032592 | 0.203 |
| GCST90032593 | 0.027 |
| GCST90032594 | 0.558 |
| GCST90032595 | 0.731 |
| GCST90032596 | 0.112 |
| GCST90032597 | 0.209 |
| GCST90032598 | 0.675 |
| GCST90032599 | 0.195 |
| GCST90032600 | 0.632 |
| GCST90032601 | 0.023 |
| GCST90032602 | 0.567 |
| GCST90032603 | 0.41 |
| GCST90032604 | 0.43 |
| GCST90032605 | 0.497 |
| GCST90032606 | 0.269 |
| GCST90032607 | 0.851 |
| GCST90032608 | 0.688 |
| GCST90032609 | 0.874 |
| GCST90032610 | 0.679 |
| GCST90032611 | 0.322 |
| GCST90032612 | 0.317 |
| GCST90032613 | 0.455 |
| GCST90032614 | 0.294 |
| GCST90032615 | 0.047 |
| GCST90032616 | 0.093 |
| GCST90032617 | 0.307 |
| GCST90032618 | 0.538 |
| GCST90032619 | 0.35 |
| GCST90032620 | 0.824 |
| GCST90032621 | 0.025 |
| GCST90032622 | 0.506 |
| GCST90032623 | 0.186 |
| GCST90032624 | 0.897 |
| GCST90032625 | 0.542 |
| GCST90032626 | 0.491 |
| GCST90032627 | 0.031 |
| GCST90032628 | 0.98 |
| GCST90032629 | 0.381 |
| GCST90032630 | 0.325 |
| GCST90032631 | 0.624 |
| GCST90032632 | 0.254 |

| | |
|---|---|
| GCST90032633 | 0.013 |
| GCST90032634 | 0.137 |
| GCST90032635 | 0.292 |
| GCST90032636 | 0.419 |
| GCST90032637 | 0.137 |
| GCST90032638 | 0.731 |
| GCST90032639 | 0.374 |
| GCST90032640 | 0.293 |
| GCST90032641 | 0.925 |
| GCST90032642 | 0.042 |
| GCST90032643 | 0.959 |
| GCST90032644 | 0.664 |
