# Supplementary material for: Two-sample Mendelian randomization study of gut microbiota and inflammatory proteins: Predictive, preventive, and personalized treatment for migraine
Source: Neural Regen Res. 2026 Feb 5;21(9):4474–80. doi: 10.4103/NRR.NRR-D-25-01711 (PMC13557522; doi:10.4103/NRR.NRR-D-25-01711)
Supplement: Supplementary file 2 [file NRR-21-4474_Suppl2.pdf]

Additional Table 5 MR-PRESSO results of associations between inflammatory proteins and migraine

	Exposure	MR Analysis	Causal Estimate	Sd	T-stat	P-value	RSSobs	Pvalue	phe
1	beta.exposure	Raw	8	0.0296089531363614	-1.086542006	0.302735392395825	6.98754100889589	0.831	level of eukaryotic translation initiation factor 4E-binding protein 1 in blood plasma
2	beta.exposure	Outlier-corrected	NA	NA	NA	NA	6.98754100889589	0.831	level of eukaryotic translation initiation factor 4E-binding protein 1 in blood plasma
3	beta.exposure	Raw	-0.03588607	0.0160431033837163	-2.23685337	0.0434486758730284	11.0402375064597	0.754	adenosine deaminase measurement
4	beta.exposure	Outlier-corrected	NA	NA	NA	NA	11.0402375064597	0.754	adenosine deaminase measurement
5	beta.exposure	Raw	-0.04221077	0.053086053669897	-0.795138583	0.438169959712403	43.5248940354302	0.001	artemin measurement
6	beta.exposure	Outlier-corrected	-0.009768995	0.0322591416244727	-0.302828743	0.766811687485823	43.5248940354302	0.001	artemin measurement
7	beta.exposure	Raw	0.0871262150895467	0.0274204324496632	3.17741943893437	0.0155463889193838	2.67392525556636	0.966	axin-1 measurement
8	beta.exposure	Outlier-corrected	NA	NA	NA	NA	2.67392525556636	0.966	axin-1 measurement
9	beta.exposure	Raw	0.108004249581672	0.0417786049962005	2.58515691444207	0.0176852068435776	31.0180461450213	0.145	beta-nerve growth factor amount
10	beta.exposure	Outlier-corrected	NA	NA	NA	NA	31.0180461450213	0.145	beta-nerve growth factor amount

11	beta.exposure	Raw	-0.010290906	0.0308784429675114	-0.333271526	0.744243262942736	12.9010625474406	0.641	caspase-8 measurement
12	beta.exposure	Outlier-corrected	NA	NA	NA	NA	12.9010625474406	0.641	caspase-8 measurement
13	beta.exposure	Raw	-0.041017763	0.0376396967569626	-1.089747432	0.290199063595989	33.994283382494	0.053	eotaxin measurement
14	beta.exposure	Outlier-corrected	NA	NA	NA	NA	33.994283382494	0.053	eotaxin measurement
15	beta.exposure	Raw	-0.041232417	0.0247593819301695	-1.66532499	0.112251137305983	27.1489239589998	0.212	C-C motif chemokine 19 level
16	beta.exposure	Outlier-corrected	NA	NA	NA	NA	27.1489239589998	0.212	C-C motif chemokine 19 level
17	beta.exposure	Raw	-0.025741159	0.0385112993047811	-0.668405359	0.511155654354065	31.2785194953036	0.12	C-C motif chemokine 20 measurement
18	beta.exposure	Outlier-corrected	NA	NA	NA	NA	31.2785194953036	0.12	C-C motif chemokine 20 measurement
19	beta.exposure	Raw	0.0198016905185718	0.0282549284869426	0.700822531818573	0.491106892655795	52.4793475180691	0.044	C-C motif chemokine 23 measurement
20	beta.exposure	Outlier-corrected	-0.041163906	0.0412151441643149	-0.99875682	0.329843836515453	52.4793475180691	0.044	C-C motif chemokine 23 measurement
21	beta.exposure	Raw	-0.025487051	0.0146306057719078	-1.742036624	0.0948612864992292	33.0556159257722	0.193	C-C motif chemokine 25 measurement
22	beta.exposure	Outlier-corrected	NA	NA	NA	NA	33.0556159257722	0.193	C-C motif chemokine 25 measurement
23	beta.exposure	Raw	0.0479355234594305	0.0404089302831971	1.18626063900937	0.250142055595833	25.8653276937275	0.262	C-C motif chemokine 28 measurement
24	beta.exposure	Outlier-corrected	NA	NA	NA	NA	25.8653276937275	0.262	C-C motif chemokine 28 measurement

25	beta.exposure	Raw	-0.027429101	0.0132338449263474	-2.072647943	0.0537238503118945	13.3328239441177	0.816	C-C motif chemokine 4-like measurement
26	beta.exposure	Outlier-corrected	NA	NA	NA	NA	13.3328239441177	0.816	C-C motif chemokine 4-like measurement
27	beta.exposure	Raw	0.0189666045197734	0.0346291152425759	0.547706875757954	0.589954096204072	39.6999495996012	0.023	natural killer cell receptor 2B4 measurement
28	beta.exposure	Outlier-corrected	NA	NA	NA	NA	39.6999495996012	0.023	natural killer cell receptor 2B4 measurement
29	beta.exposure	Raw	-0.022417742	0.0189634695099252	-1.182154046	0.256834543472939	14.0688802327808	0.645	CD40 measurement
30	beta.exposure	Outlier-corrected	NA	NA	NA	NA	14.0688802327808	0.645	CD40 measurement
31	beta.exposure	Raw	0.0795147998011288	0.02218258061599	3.58456038896627	0.00185362642247493	14.3253425265193	0.878	t-cell surface glycoprotein CD5 measurement
32	beta.exposure	Outlier-corrected	NA	NA	NA	NA	14.3253425265193	0.878	t-cell surface glycoprotein CD5 measurement
33	beta.exposure	Raw	0.0352131961677531	0.0152284775742676	2.31232544395999	0.0335505772952985	16.9050051231444	0.67	level of T-cell differentiation antigen CD6 in blood plasma
34	beta.exposure	Outlier-corrected	NA	NA	NA	NA	16.9050051231444	0.67	level of T-cell differentiation antigen CD6 in blood plasma

35	beta.exposure	Raw	0.0310204512818762	0.027824593473969	1.11485730459628	0.275951394896174	48.4150847977679	0.004	CUB domain-containing protein 1 measurement
36	beta.exposure	Outlier-corrected	0.0299103420097966	0.0196415303283736	1.52281118170252	0.142051684167628	48.4150847977679	0.004	CUB domain-containing protein 1 measurement
37	beta.exposure	Raw	-0.013546398	0.0391391876413689	-0.346108312	0.734068134366762	23.9236909786267	0.138	macrophage colony-stimulating factor 1 level
38	beta.exposure	Outlier-corrected	NA	NA	NA	NA	23.9236909786267	0.138	macrophage colony-stimulating factor 1 level
39	beta.exposure	Raw	0.00272563605343862	0.0137525721040636	0.198191002585855	0.844278433480609	24.4315827968732	0.797	cystatin-D measurement
40	beta.exposure	Outlier-corrected	NA	NA	NA	NA	24.4315827968732	0.797	cystatin-D measurement
41	beta.exposure	Raw	0.0160720938061458	0.0510635381725908	0.314746967823174	0.756213849450996	54.026632358002	<0.001	fractalkine measurement
42	beta.exposure	Outlier-corrected	0.0257463252797245	0.0340336133724132	0.756496966631049	0.459138943923497	54.026632358002	<0.001	fractalkine measurement
43	beta.exposure	Raw	0.020554003309703	0.0443236232722294	0.463725701833157	0.655200410521084	25.4694362900372	0.221	CXCL1 measurement
44	beta.exposure	Outlier-corrected	NA	NA	NA	NA	25.4694362900372	0.221	CXCL1 measurement
45	beta.exposure	Raw	0.0558268724017349	0.028268206002639	1.97489972998368	0.0609605520592104	27.208061981244	0.329	C-X-C motif chemokine 10 measurement
46	beta.exposure	Outlier-corrected	NA	NA	NA	NA	27.208061981244	0.329	C-X-C motif chemokine 10 measurement
47	beta.exposure	Raw	0.0762499089145559	0.0319810918094785	2.38421844284744	0.0266270552104123	30.1583785159268	0.175	C-X-C motif chemokine 11 measurement
48	beta.exposure	Outlier-corrected	NA	NA	NA	NA	30.1583785159268	0.175	C-X-C motif chemokine 11 measurement

49	beta.exposure	Raw	0.0268649680673046	0.0368572758925917	0.728891851519187	0.478994197604993	56.78406742	0.007	C-X-C motif chemokine 5 measurement
50	beta.exposure	Outlier-corrected	0.0539987812421418	0.0483728419131996	1.11630367591463	0.290396787179863	56.78406742	0.007	C-X-C motif chemokine 5 measurement
51	beta.exposure	Raw	0.00846097238963098	0.0478715935928187	0.176743069420196	0.862658133977283	21.5493778700861	0.111	C-X-C motif chemokine 6 level
52	beta.exposure	Outlier-corrected	NA	NA	NA	NA	21.5493778700861	0.111	C-X-C motif chemokine 6 level
53	beta.exposure	Raw	0.00288865651369964	0.0316159417345766	0.0913670874633628	0.928109846650413	25.4817321113184	0.29	C-X-C motif chemokine 9 measurement
54	beta.exposure	Outlier-corrected	NA	NA	NA	NA	25.4817321113184	0.29	C-X-C motif chemokine 9 measurement
55	beta.exposure	Raw	0.00521439752952632	0.0268160439803466	0.194450662944464	0.848432078060213	13.9231463085425	0.662	delta and Notch-like epidermal growth factor-related receptor measurement
56	beta.exposure	Outlier-corrected	NA	NA	NA	NA	13.9231463085425	0.662	delta and Notch-like epidermal growth factor-related receptor measurement
57	beta.exposure	Raw	0.0111308611592942	0.0300151191690865	0.370841811308157	0.716306472884007	12.8076650740015	0.688	protein S100-A12 measurement
58	beta.exposure	Outlier-corrected	NA	NA	NA	NA	12.8076650740015	0.688	protein S100-A12 measurement

59	beta.exposure	Raw	-0.00197999	0.0373967728808793	-0.052945489	0.958300619752977	40.710079985913	0.015	fibroblast growth factor 19 amount
60	beta.exposure	Outlier-corrected	0.0127714484730682	0.0335168828762613	0.381045233836876	0.707396982201266	40.710079985913	0.015	fibroblast growth factor 19 amount
61	beta.exposure	Raw	0.0592247369995924	0.0282625110664667	2.09552282386763	0.0562678594333279	14.6581245594332	0.505	fibroblast growth factor 21 amount
62	beta.exposure	Outlier-corrected	NA	NA	NA	NA	14.6581245594332	0.505	fibroblast growth factor 21 amount
63	beta.exposure	Raw	0.0109784067799795	0.0482251679838865	0.227648906970065	0.823210242645456	27.4694248290263	0.049	fibroblast growth factor 23 amount
64	beta.exposure	Outlier-corrected	NA	NA	NA	NA	27.4694248290263	0.049	fibroblast growth factor 23 amount
65	beta.exposure	Raw	0.0256918316478899	0.0126735044626652	2.0272081588463	0.0549271759456533	15.8370536536482	0.896	fibroblast growth factor 5 amount
66	beta.exposure	Outlier-corrected	NA	NA	NA	NA	15.8370536536482	0.896	fibroblast growth factor 5 amount
67	beta.exposure	Raw	-0.023053978	0.0272707648754262	-0.845373359	0.405068752034921	46.8959796939157	0.035	fms-related tyrosine kinase 3 ligand measurement
68	beta.exposure	Outlier-corrected	-0.032330915	0.0244847309802715	-1.320452122	0.19776861734708	46.8959796939157	0.035	fms-related tyrosine kinase 3 ligand measurement
69	beta.exposure	Raw	-0.015676924	0.0276904620658412	-0.566148882	0.582658213226292	11.4172152656771	0.588	glial cell line-derived neurotrophic factor measurement

70	beta.exposure	Outlier-corrected	NA	NA	NA	NA	11.4172152656771	0.588	glial cell line-derived neurotrophic factor measurement
71	beta.exposure	Raw	0.0442090445037499	0.0374739130362172	1.17972853438133	0.254354488675753	19.7794478284454	0.411	hepatocyte growth factor amount
72	beta.exposure	Outlier-corrected	NA	NA	NA	NA	19.7794478284454	0.411	hepatocyte growth factor amount
73	beta.exposure	Raw	-0.013147049	0.046605737850963	-0.282090774	0.784253336723115	14.537673779119	0.243	interferon gamma measurement
74	beta.exposure	Outlier-corrected	NA	NA	NA	NA	14.537673779119	0.243	interferon gamma measurement
75	beta.exposure	Raw	0.0290542819707794	0.0282636574129089	1.02797318642524	0.318367636647584	18.1044345851574	0.531	interleukin-10 measurement
76	beta.exposure	Outlier-corrected	NA	NA	NA	NA	18.1044345851574	0.531	interleukin-10 measurement
77	beta.exposure	Raw	0.0373863203450418	0.0352908024186548	1.0593785854322	0.317029708388132	12.6525080285795	0.381	interleukin-10 receptor subunit alpha measurement
78	beta.exposure	Outlier-corrected	NA	NA	NA	NA	12.6525080285795	0.381	interleukin-10 receptor subunit alpha measurement
79	beta.exposure	Raw	-0.000237912	0.0197072445663882	-0.012072321	0.990508460902359	22.3812327471565	0.37	interleukin-10 receptor subunit beta measurement
80	beta.exposure	Outlier-corrected	NA	NA	NA	NA	22.3812327471565	0.37	interleukin-10 receptor subunit beta measurement

81	beta.exposure	Raw	0.0231462809266733	0.0237177836120401	0.975904043366147	0.337775279755131	75.153067019151	<0.001	level of interleukin-12 subunit beta in blood
82	beta.exposure	Outlier-corrected	0.0227248347329177	0.0196922754720566	1.15399740193378	0.259414305970787	75.153067019151	<0.001	level of interleukin-12 subunit beta in blood
83	beta.exposure	Raw	-0.021448602	0.034045220183168	-0.630003334	0.542818835795518	11.0172363677903	0.543	interleukin-13 measurement
84	beta.exposure	Outlier-corrected	NA	NA	NA	NA	11.0172363677903	0.543	interleukin-13 measurement
85	beta.exposure	Raw	-0.029962262	0.0217349977800958	-1.378526095	0.195430293254108	14.1351046969275	0.431	interleukin-15 receptor subunit alpha measurement
86	beta.exposure	Outlier-corrected	NA	NA	NA	NA	14.1351046969275	0.431	interleukin-15 receptor subunit alpha measurement
87	beta.exposure	Raw	0.0191933136756423	0.0458977813477166	0.418175195228628	0.683866299814005	16.8184893408487	0.211	Interleukin-17A measurement
88	beta.exposure	Outlier-corrected	NA	NA	NA	NA	16.8184893408487	0.211	Interleukin-17A measurement
89	beta.exposure	Raw	0.00531156907321374	0.0357716694469299	0.148485356018787	0.883815029267871	19.8876636804528	0.374	interleukin-17C measurement
90	beta.exposure	Outlier-corrected	NA	NA	NA	NA	19.8876636804528	0.374	interleukin-17C measurement
91	beta.exposure	Raw	-0.044355512	0.0343287106800658	-1.292082069	0.211059824018434	43.853413741802	0.015	interleukin 18 measurement
92	beta.exposure	Outlier-corrected	NA	NA	NA	NA	43.853413741802	0.015	interleukin 18 measurement
93	beta.exposure	Raw	0.0181988726843018	0.0226491015651667	0.803514109905839	0.431615871064263	43.3201863677404	0.147	interleukin-18 receptor 1 measurement

94	beta.exposure	Outlier-corrected	NA	NA	NA	NA	43.3201863677404	0.147		interleukin-18 receptor 1 measurement
95	beta.exposure	Raw	-0.006223736	0.0520106697619429	-0.11966267	0.906907900942031	26.6631680126412	0.023		interleukin-1 alpha measurement
96	beta.exposure	Outlier-corrected	NA	NA	NA	NA	26.6631680126412	0.023		interleukin-1 alpha measurement
97	beta.exposure	Raw	0.0676259555011003	0.0493281739685637	1.37093977052947	0.19197197832385	30.4767915935223	0.026		interleukin-2 measurement
98	beta.exposure	Outlier-corrected	NA	NA	NA	NA	30.4767915935223	0.026		interleukin-2 measurement
99	beta.exposure	Raw	-0.014016436	0.0509210336982601	-0.275258276	0.788721541129575	16.2824573357874	0.211		interleukin-20 measurement
100	beta.exposure	Outlier-corrected	NA	NA	NA	NA	16.2824573357874	0.211		interleukin-20 measurement
101	beta.exposure	Raw	0.0337602266999459	0.0437959677208514	0.770852397077471	0.458620162084937	16.663399884289	0.191		interleukin-20 receptor subunit alpha measurement
102	beta.exposure	Outlier-corrected	NA	NA	NA	NA	16.663399884289	0.191		interleukin-20 receptor subunit alpha measurement
103	beta.exposure	Raw	0.0162419339971396	0.0658524236897679	0.246641400378149	0.810175028923724	22.4936265710968	0.051		interleukin-22 receptor subunit alpha-1 measurement
104	beta.exposure	Outlier-corrected	NA	NA	NA	NA	22.4936265710968	0.051		interleukin-22 receptor subunit alpha-1 measurement
105	beta.exposure	Raw	0.0141511673708128	0.060187755885004	0.235117046029267	0.819380239346495	15.8978710169244	0.193		interleukin-24 measurement
106	beta.exposure	Outlier-corrected	NA	NA	NA	NA	15.8978710169244	0.193		interleukin-24 measurement

107	beta.exposure	Raw	0.0492260509030353	0.0433284910133787	1.13611274594898	0.280055763666827	11.907722074541	0.561	interleukin-2 receptor subunit beta measurement
108	beta.exposure	Outlier-corrected	NA	NA	NA	NA	11.907722074541	0.561	interleukin-2 receptor subunit beta measurement
109	beta.exposure	Raw	0.000193560980257567	0.0357077526669133	0.00542069903035146	0.995781543845747	9.66052756128646	0.63	level of interleukin-33 in blood plasma
110	beta.exposure	Outlier-corrected	NA	NA	NA	NA	9.66052756128646	0.63	level of interleukin-33 in blood plasma
111	beta.exposure	Raw	0.123461190398692	0.0557927983793417	2.21285172970291	0.0578232537595618	15.1971044141112	0.197	interleukin-4 measurement
112	beta.exposure	Outlier-corrected	NA	NA	NA	NA	15.1971044141112	0.197	interleukin-4 measurement
113	beta.exposure	Raw	-0.01328875	0.0407772470149735	-0.325886405	0.75122726338575	14.2598235129935	0.343	interleukin-5 measurement
114	beta.exposure	Outlier-corrected	NA	NA	NA	NA	14.2598235129935	0.343	interleukin-5 measurement
115	beta.exposure	Raw	0.0920172946893128	0.0496335583711904	1.85393305878154	0.106152619947473	16.163673921107	0.227	interleukin-6 measurement
116	beta.exposure	Outlier-corrected	NA	NA	NA	NA	16.163673921107	0.227	interleukin-6 measurement
117	beta.exposure	Raw	-0.030122048	0.0550753523339262	-0.546924298	0.595351607624654	18.3437015032999	0.18	interleukin-7 measurement
118	beta.exposure	Outlier-corrected	NA	NA	NA	NA	18.3437015032999	0.18	interleukin-7 measurement
119	beta.exposure	Raw	0.0617882659374243	0.0383910253080339	1.60944557853458	0.127069163940603	19.1943216270809	0.406	interleukin-8 measurement
120	beta.exposure	Outlier-corrected	NA	NA	NA	NA	19.1943216270809	0.406	interleukin-8 measurement
121	beta.exposure	Raw	-0.006268168	0.0284624332439224	-0.220226001	0.828173585609835	17.8874865003587	0.601	transforming growth factor beta-1 amount

122	beta.exposure	Outlier-corrected	NA	NA	NA	NA	17.8874865003587	0.601	transforming growth factor beta-1 amount
123	beta.exposure	Raw	-0.035721322	0.0502733288075638	-0.710542207	0.493605828694785	15.9621730462987	0.217	leukemia inhibitory factor measurement
124	beta.exposure	Outlier-corrected	NA	NA	NA	NA	15.9621730462987	0.217	leukemia inhibitory factor measurement
125	beta.exposure	Raw	-0.030557719	0.0351980487738808	-0.868165134	0.398981222295328	42.9038586876986	0.021	leukemia inhibitory factor receptor measurement
126	beta.exposure	Outlier-corrected	0.013794899022179	0.0342697443590547	0.402538719800345	0.693828504072893	42.9038586876986	0.021	leukemia inhibitory factor receptor measurement
127	beta.exposure	Raw	-0.038401212	0.0197189632891583	-1.947425503	0.064978878796563	20.5120049634658	0.609	C-C motif chemokine 2 level
128	beta.exposure	Outlier-corrected	NA	NA	NA	NA	20.5120049634658	0.609	C-C motif chemokine 2 level
129	beta.exposure	Raw	-0.012551985	0.0128906778332414	-0.973725786	0.338836288163155	28.2782423969389	0.535	monocyte chemotactic protein-2 measurement
130	beta.exposure	Outlier-corrected	NA	NA	NA	NA	28.2782423969389	0.535	monocyte chemotactic protein-2 measurement
131	beta.exposure	Raw	-0.054869015	0.0264965416566933	-2.070799102	0.0549123590990827	15.1169375309677	0.647	C-C motif chemokine 7 level

132	beta.exposure	Outlier-corrected	NA	NA	NA	NA	15.1169375309677	0.647	C-C motif chemokine 7 level
133	beta.exposure	Raw	0.0309295099237766	0.0234422899430923	1.3193894452658	0.204533854281818	21.1941326379083	0.335	C-C motif chemokine 13 level
134	beta.exposure	Outlier-corrected	NA	NA	NA	NA	21.1941326379083	0.335	C-C motif chemokine 13 level
135	beta.exposure	Raw	0.013871392791141	0.0311478214568815	0.445340705780769	0.663399210742358	29.091131487498	0.059	C-C motif chemokine 3 level
136	beta.exposure	Outlier-corrected	NA	NA	NA	NA	29.091131487498	0.059	C-C motif chemokine 3 level
137	beta.exposure	Raw	-0.033510079	0.0439764517997474	-0.762000523	0.457874593868374	36.3987157038224	0.004	matrix metalloproteinase 1 measurement
138	beta.exposure	Outlier-corrected	-0.008266204	0.040295545174941	-0.205139401	0.840415918318687	36.3987157038224	0.004	matrix metalloproteinase 1 measurement
139	beta.exposure	Raw	0.0159418985058058	0.030107336721558	0.529502116153331	0.605382090103389	19.044222767675	0.292	matrix metalloproteinase 10 measurement
140	beta.exposure	Outlier-corrected	NA	NA	NA	NA	19.044222767675	0.292	matrix metalloproteinase 10 measurement
141	beta.exposure	Raw	-0.001242311	0.0371327987008752	-0.033455907	0.973819255351663	21.7899216202518	0.147	level of neurturin in blood plasma
142	beta.exposure	Outlier-corrected	NA	NA	NA	NA	21.7899216202518	0.147	level of neurturin in blood plasma
143	beta.exposure	Raw	0.0140619144420645	0.0381827593406585	0.368279157527803	0.717492207559885	20.2798142108141	0.329	neurotrophin-3 measurement

144	beta.exposure	Outlier-corrected	NA	NA	NA	NA	20.2798142108141	0.329	neurotrophin-3 measurement
145	beta.exposure	Raw	0.00140849629945139	0.0393057884272954	0.0358343225211399	0.971831881328438	33.4828026165922	0.024	tumor necrosis factor receptor superfamily member 11B amount
146	beta.exposure	Outlier-corrected	-0.018876054	0.0265210035099903	-0.71173982	0.486874634541139	33.4828026165922	0.024	tumor necrosis factor receptor superfamily member 11B amount
147	beta.exposure	Raw	0.100905155454646	0.0373917888892469	2.69859128038842	0.0173043199956028	21.0257513061211	0.224	oncostatin-M measurement
148	beta.exposure	Outlier-corrected	NA	NA	NA	NA	21.0257513061211	0.224	oncostatin-M measurement
149	beta.exposure	Raw	0.0221499725559535	0.037339011177038	0.593212617520382	0.561877407560876	20.4866695068121	0.284	programmed cell death 1 ligand 1 amount
150	beta.exposure	Outlier-corrected	NA	NA	NA	NA	20.4866695068121	0.284	programmed cell death 1 ligand 1 amount
151	beta.exposure	Raw	0.00641975589917386	0.0234689474263258	0.273542557429424	0.786250486708512	51.6914203386904	0.015	kit ligand amount
152	beta.exposure	Outlier-corrected	-0.018582147	0.0185114858962408	-1.003817151	0.323769572500067	51.6914203386904	0.015	kit ligand amount
153	beta.exposure	Raw	-0.056768413	0.0440449049939278	-1.288875817	0.221734281055329	18.7851320959627	0.198	SIR2-like protein 2 measurement
154	beta.exposure	Outlier-corrected	NA	NA	NA	NA	18.7851320959627	0.198	SIR2-like protein 2 measurement

155	beta.exposure	Raw	0.0720579978928067	0.0307748857339588	2.34145460411226	0.027843306966856	35.3263796556312	0.135	signaling lymphocytic activation molecule measurement
156	beta.exposure	Outlier-corrected	NA	NA	NA	NA	35.3263796556312	0.135	signaling lymphocytic activation molecule measurement
157	beta.exposure	Raw	0.031967198517078	0.0222127938012802	1.4391345277439	0.164189482008862	22.75775573	0.545	sulfotrasferase 1A1 measurement
158	beta.exposure	Outlier-corrected	NA	NA	NA	NA	22.75775573	0.545	sulfotrasferase 1A1 measurement
159	beta.exposure	Raw	-0.021998161	0.0687924046862936	-0.319776014	0.755718888769203	23.9847495827003	0.033	STAM-binding protein amount
160	beta.exposure	Outlier-corrected	NA	NA	NA	NA	23.9847495827003	0.033	STAM-binding protein amount
161	beta.exposure	Raw	0.0159637735419128	0.0500695591410909	0.318831917351788	0.754918777786677	30.8743666175903	0.019	protransforming growth factor alpha amount
162	beta.exposure	Outlier-corrected	NA	NA	NA	NA	30.8743666175903	0.019	protransforming growth factor alpha amount
163	beta.exposure	Raw	-0.00567076	0.0375762574448273	-0.150913378	0.881820317571821	25.4954840098297	0.154	tumor necrosis factor amount
164	beta.exposure	Outlier-corrected	NA	NA	NA	NA	25.4954840098297	0.154	tumor necrosis factor amount
165	beta.exposure	Raw	-0.011245653	0.0196828814843525	-0.571341799	0.573559157441299	46.2367284053083	0.095	lymphotoxin-alpha amount

166	beta.exposure	Outlier-corrected	NA	NA	NA	NA	46.2367284053083	0.095	lymphotoxin-alpha amount
167	beta.exposure	Raw	0.024583356391633	0.0273205258438291	0.899812709761062	0.376478818561468	34.9198655442603	0.17	tumor necrosis factor receptor superfamily member 9 amount
168	beta.exposure	Outlier-corrected	NA	NA	NA	NA	34.9198655442603	0.17	tumor necrosis factor receptor superfamily member 9 amount
169	beta.exposure	Raw	-0.01233661	0.0229344861412724	-0.537906532	0.595595084032628	24.8274290570083	0.543	tumor necrosis factor ligand superfamily member 14 measurement
170	beta.exposure	Outlier-corrected	NA	NA	NA	NA	24.8274290570083	0.543	tumor necrosis factor ligand superfamily member 14 measurement
171	beta.exposure	Raw	-0.00269399	0.0176320663431105	-0.152789258	0.879744047631485	30.6976752067678	0.351	TNF-related apoptosis-inducing ligand measurement
172	beta.exposure	Outlier-corrected	NA	NA	NA	NA	30.6976752067678	0.351	TNF-related apoptosis-inducing ligand measurement

173	beta.exposure	Raw	0.021127948625745	0.0178218166745843	1.18551037818022	0.246144903711441	19.9108821820081	0.878	TNF-related activation-induced cytokine measurement
174	beta.exposure	Outlier-corrected	NA	NA	NA	NA	19.9108821820081	0.878	TNF-related activation-induced cytokine measurement
175	beta.exposure	Raw	0.0158966950210539	0.0425863835067426	0.373281169051065	0.714530574186981	20.5594237507777	0.209	thymic stromal lymphopoietin measurement
176	beta.exposure	Outlier-corrected	NA	NA	NA	NA	20.5594237507777	0.209	thymic stromal lymphopoietin measurement
177	beta.exposure	Raw	-0.027929972	0.0257527619656009	-1.084542769	0.288472784515536	28.1589196063378	0.411	tumor necrosis factor ligand superfamily member 12 amount
178	beta.exposure	Outlier-corrected	NA	NA	NA	NA	28.1589196063378	0.411	tumor necrosis factor ligand superfamily member 12 amount
179	beta.exposure	Raw	0.0184875947359031	0.0267298738425004	0.691645416840982	0.497111279899337	25.8692798728883	0.273	urokinase-type plasminogen activator measurement

180	beta.exposure	Outlier-corrected	NA	NA	NA	NA	25.8692798728883	0.273	urokinase-type plasminogen activator measurement
181	beta.exposure	Raw	-0.012081426	0.0233663664371802	-0.517043439	0.610062581274436	46.3908903673883	0.065	vascular endothelial growth factor A amount
182	beta.exposure	Outlier-corrected	NA	NA	NA	NA	46.3908903673883	0.065	vascular endothelial growth factor A amount
