# Supplementary material for: Two-sample Mendelian randomization study of gut microbiota and inflammatory proteins: Predictive, preventive, and personalized treatment for migraine
Source: Neural Regen Res. 2026 Feb 5;21(9):4474–80. doi: 10.4103/NRR.NRR-D-25-01711 (PMC13557522; doi:10.4103/NRR.NRR-D-25-01711)
Supplement: Supplementary file 3 [file NRR-21-4474_Suppl3.pdf]

Additional Table 6 MR-PRESSO results of associations between gut microbiota and inflammatory proteins

FolderName	Line12	Line13
GCST90032179+GCST90274762	[[1]]$`MR-PRESSO results`$`Global Test`$Pvalue	[1] 0.966
GCST90032179+GCST90274773	[[1]]$`MR-PRESSO results`$`Global Test`$Pvalue	[1] 0.571
GCST90032179+GCST90274774	[[1]]$`MR-PRESSO results`$`Global Test`$Pvalue	[1] 0.263
GCST90032179+GCST90274780	[[1]]$`MR-PRESSO results`$`Global Test`$Pvalue	[1] 0.811
GCST90032179+GCST90274781	[[1]]$`MR-PRESSO results`$`Global Test`$Pvalue	[1] 0.853
GCST90032179+GCST90274813	[[1]]$`MR-PRESSO results`$`Global Test`$Pvalue	[1] 0.102
GCST90032179+GCST90274831	[[1]]$`MR-PRESSO results`$`Global Test`$Pvalue	[1] 0.157
GCST90032179+GCST90274835	[[1]]$`MR-PRESSO results`$`Global Test`$Pvalue	[1] 0.94
GCST90032181+GCST90274762	[[1]]$`MR-PRESSO results`$`Global Test`$Pvalue	[1] 0.229
GCST90032181+GCST90274773	[[1]]$`MR-PRESSO results`$`Global Test`$Pvalue	[1] 0.155
GCST90032181+GCST90274774	[[1]]$`MR-PRESSO results`$`Global Test`$Pvalue	[1] 0.057
GCST90032181+GCST90274780	[[1]]$`MR-PRESSO results`$`Global Test`$Pvalue	[1] 0.505
GCST90032181+GCST90274781	[[1]]$`MR-PRESSO results`$`Global Test`$Pvalue	[1] 0.274
GCST90032181+GCST90274813	[[1]]$`MR-PRESSO results`$`Global Test`$Pvalue	[1] 0.139
GCST90032181+GCST90274831	[[1]]$`MR-PRESSO results`$`Global Test`$Pvalue	[1] 0.106
GCST90032181+GCST90274835	[[1]]$`MR-PRESSO results`$`Global Test`$Pvalue	[1] 0.347
GCST90032188+GCST90274762	[[1]]$`MR-PRESSO results`$`Global Test`$Pvalue	[1] 0.569
GCST90032188+GCST90274773	[[1]]$`MR-PRESSO results`$`Global Test`$Pvalue	[1] 0.574
GCST90032188+GCST90274774	[[1]]$`MR-PRESSO results`$`Global Test`$Pvalue	[1] 0.615
GCST90032188+GCST90274780	[[1]]$`MR-PRESSO results`$`Global Test`$Pvalue	[1] 0.526
GCST90032188+GCST90274781	[[1]]$`MR-PRESSO results`$`Global Test`$Pvalue	[1] 0.398
GCST90032188+GCST90274813	[[1]]$`MR-PRESSO results`$`Global Test`$Pvalue	[1] 0.4
GCST90032188+GCST90274831	[[1]]$`MR-PRESSO results`$`Global Test`$Pvalue	[1] 0.528
GCST90032188+GCST90274835	[[1]]$`MR-PRESSO results`$`Global Test`$Pvalue	[1] 0.771
GCST90032202+GCST90274762	[[1]]$`MR-PRESSO results`$`Global Test`$Pvalue	[1] 0.56
GCST90032202+GCST90274773	[[1]]$`MR-PRESSO results`$`Global Test`$Pvalue	[1] 0.182
GCST90032202+GCST90274774	[[1]]$`MR-PRESSO results`$`Global Test`$Pvalue	[1] 0.197
GCST90032202+GCST90274780	[[1]]$`MR-PRESSO results`$`Global Test`$Pvalue	[1] 0.885

GCST90032202+GCST90274781	[[1]]$`MR-PRESSO results`$`Global Test`$Pvalue	[1] 0.996
GCST90032202+GCST90274813	[[1]]$`MR-PRESSO results`$`Global Test`$Pvalue	[1] 0.975
GCST90032202+GCST90274831	[[1]]$`MR-PRESSO results`$`Global Test`$Pvalue	[1] 0.992
GCST90032202+GCST90274835	[[1]]$`MR-PRESSO results`$`Global Test`$Pvalue	[1] 0.25
GCST90032205+GCST90274762	[[1]]$`MR-PRESSO results`$`Global Test`$Pvalue	[1] 0.436
GCST90032205+GCST90274773	[[1]]$`MR-PRESSO results`$`Global Test`$Pvalue	[1] 0.652
GCST90032205+GCST90274774	[[1]]$`MR-PRESSO results`$`Global Test`$Pvalue	[1] 0.714
GCST90032205+GCST90274780	[[1]]$`MR-PRESSO results`$`Global Test`$Pvalue	[1] 0.569
GCST90032205+GCST90274781	[[1]]$`MR-PRESSO results`$`Global Test`$Pvalue	[1] 0.865
GCST90032205+GCST90274813	[[1]]$`MR-PRESSO results`$`Global Test`$Pvalue	[1] 0.729
GCST90032205+GCST90274831	[[1]]$`MR-PRESSO results`$`Global Test`$Pvalue	[1] 0.501
GCST90032205+GCST90274835	[[1]]$`MR-PRESSO results`$`Global Test`$Pvalue	[1] 0.541
GCST90032219+GCST90274762	[[1]]$`MR-PRESSO results`$`Global Test`$Pvalue	[1] 0.753
GCST90032219+GCST90274773	[[1]]$`MR-PRESSO results`$`Global Test`$Pvalue	[1] 0.409
GCST90032219+GCST90274774	[[1]]$`MR-PRESSO results`$`Global Test`$Pvalue	[1] 0.456
GCST90032219+GCST90274780	[[1]]$`MR-PRESSO results`$`Global Test`$Pvalue	[1] 0.03
GCST90032219+GCST90274781	[[1]]$`MR-PRESSO results`$`Global Test`$Pvalue	[1] 0.587
GCST90032219+GCST90274813	[[1]]$`MR-PRESSO results`$`Global Test`$Pvalue	[1] 0.334
GCST90032219+GCST90274831	[[1]]$`MR-PRESSO results`$`Global Test`$Pvalue	[1] 0.298
GCST90032219+GCST90274835	[[1]]$`MR-PRESSO results`$`Global Test`$Pvalue	[1] 0.634
GCST90032223+GCST90274762	[[1]]$`MR-PRESSO results`$`Global Test`$Pvalue	[1] 0.349
GCST90032223+GCST90274773	[[1]]$`MR-PRESSO results`$`Global Test`$Pvalue	[1] 0.111
GCST90032223+GCST90274774	[[1]]$`MR-PRESSO results`$`Global Test`$Pvalue	[1] 0.071
GCST90032223+GCST90274780	[[1]]$`MR-PRESSO results`$`Global Test`$Pvalue	[1] 0.532
GCST90032223+GCST90274781	[[1]]$`MR-PRESSO results`$`Global Test`$Pvalue	[1] 0.397
GCST90032223+GCST90274813	[[1]]$`MR-PRESSO results`$`Global Test`$Pvalue	[1] 0.012
GCST90032223+GCST90274831	[[1]]$`MR-PRESSO results`$`Global Test`$Pvalue	[1] 0.469
GCST90032223+GCST90274835	[[1]]$`MR-PRESSO results`$`Global Test`$Pvalue	[1] 0.174
GCST90032228+GCST90274762	[[1]]$`MR-PRESSO results`$`Global Test`$Pvalue	[1] 0.294
GCST90032228+GCST90274773	[[1]]$`MR-PRESSO results`$`Global Test`$Pvalue	[1] 0.244

GCST90032228+GCST90274774	[[1]]$`MR-PRESSO results`$`Global Test`$Pvalue	[1] 0.101
GCST90032228+GCST90274780	[[1]]$`MR-PRESSO results`$`Global Test`$Pvalue	[1] 0.148
GCST90032228+GCST90274781	[[1]]$`MR-PRESSO results`$`Global Test`$Pvalue	[1] 0.252
GCST90032228+GCST90274813	[[1]]$`MR-PRESSO results`$`Global Test`$Pvalue	[1] 0.095
GCST90032228+GCST90274831	[[1]]$`MR-PRESSO results`$`Global Test`$Pvalue	[1] 0.167
GCST90032228+GCST90274835	[[1]]$`MR-PRESSO results`$`Global Test`$Pvalue	[1] 0.234
GCST90032230+GCST90274762	[[1]]$`MR-PRESSO results`$`Global Test`$Pvalue	[1] 0.258
GCST90032230+GCST90274773	[[1]]$`MR-PRESSO results`$`Global Test`$Pvalue	[1] 0.67
GCST90032230+GCST90274774	[[1]]$`MR-PRESSO results`$`Global Test`$Pvalue	[1] 0.146
GCST90032230+GCST90274780	[[1]]$`MR-PRESSO results`$`Global Test`$Pvalue	[1] 0.022
GCST90032230+GCST90274781	[[1]]$`MR-PRESSO results`$`Global Test`$Pvalue	[1] 0.515
GCST90032230+GCST90274813	[[1]]$`MR-PRESSO results`$`Global Test`$Pvalue	[1] 0.294
GCST90032230+GCST90274831	[[1]]$`MR-PRESSO results`$`Global Test`$Pvalue	[1] 0.113
GCST90032230+GCST90274835	[[1]]$`MR-PRESSO results`$`Global Test`$Pvalue	[1] 0.548
GCST90032241+GCST90274762	[[1]]$`MR-PRESSO results`$`Global Test`$Pvalue	[1] 0.167
GCST90032241+GCST90274773	[[1]]$`MR-PRESSO results`$`Global Test`$Pvalue	[1] 0.521
GCST90032241+GCST90274774	[[1]]$`MR-PRESSO results`$`Global Test`$Pvalue	[1] 0.208
GCST90032241+GCST90274780	[[1]]$`MR-PRESSO results`$`Global Test`$Pvalue	[1] 0.098
GCST90032241+GCST90274781	[[1]]$`MR-PRESSO results`$`Global Test`$Pvalue	[1] 0.339
GCST90032241+GCST90274813	[[1]]$`MR-PRESSO results`$`Global Test`$Pvalue	[1] 0.229
GCST90032241+GCST90274831	[[1]]$`MR-PRESSO results`$`Global Test`$Pvalue	[1] 0.187
GCST90032241+GCST90274835	[[1]]$`MR-PRESSO results`$`Global Test`$Pvalue	[1] 0.361
GCST90032264+GCST90274762	[[1]]$`MR-PRESSO results`$`Global Test`$Pvalue	[1] 0.453
GCST90032264+GCST90274773	[[1]]$`MR-PRESSO results`$`Global Test`$Pvalue	[1] 0.975
GCST90032264+GCST90274774	[[1]]$`MR-PRESSO results`$`Global Test`$Pvalue	[1] 0.447
GCST90032264+GCST90274780	[[1]]$`MR-PRESSO results`$`Global Test`$Pvalue	[1] 0.44
GCST90032264+GCST90274781	[[1]]$`MR-PRESSO results`$`Global Test`$Pvalue	[1] 0.215
GCST90032264+GCST90274813	[[1]]$`MR-PRESSO results`$`Global Test`$Pvalue	[1] 0.564
GCST90032264+GCST90274831	[[1]]$`MR-PRESSO results`$`Global Test`$Pvalue	[1] 0.878
GCST90032264+GCST90274835	[[1]]$`MR-PRESSO results`$`Global Test`$Pvalue	[1] 0.474

GCST90032265+GCST90274762	[[1]]$`MR-PRESSO results`$`Global Test`$Pvalue	[1] 0.349
GCST90032265+GCST90274773	[[1]]$`MR-PRESSO results`$`Global Test`$Pvalue	[1] 0.955
GCST90032265+GCST90274774	[[1]]$`MR-PRESSO results`$`Global Test`$Pvalue	[1] 0.769
GCST90032265+GCST90274780	[[1]]$`MR-PRESSO results`$`Global Test`$Pvalue	[1] 0.409
GCST90032265+GCST90274781	[[1]]$`MR-PRESSO results`$`Global Test`$Pvalue	[1] 0.129
GCST90032265+GCST90274813	[[1]]$`MR-PRESSO results`$`Global Test`$Pvalue	[1] 0.552
GCST90032265+GCST90274831	[[1]]$`MR-PRESSO results`$`Global Test`$Pvalue	[1] 0.873
GCST90032265+GCST90274835	[[1]]$`MR-PRESSO results`$`Global Test`$Pvalue	[1] 0.337
GCST90032266+GCST90274762	[[1]]$`MR-PRESSO results`$`Global Test`$Pvalue	[1] 0.019
GCST90032266+GCST90274773	[[1]]$`MR-PRESSO results`$`Global Test`$Pvalue	[1] 0.897
GCST90032266+GCST90274774	[[1]]$`MR-PRESSO results`$`Global Test`$Pvalue	[1] 0.893
GCST90032266+GCST90274780	[[1]]$`MR-PRESSO results`$`Global Test`$Pvalue	[1] 0.148
GCST90032266+GCST90274781	[[1]]$`MR-PRESSO results`$`Global Test`$Pvalue	[1] 0.155
GCST90032266+GCST90274813	[[1]]$`MR-PRESSO results`$`Global Test`$Pvalue	[1] 0.66
GCST90032266+GCST90274831	[[1]]$`MR-PRESSO results`$`Global Test`$Pvalue	[1] 0.094
GCST90032266+GCST90274835	[[1]]$`MR-PRESSO results`$`Global Test`$Pvalue	[1] 0.722
GCST90032275+GCST90274762	[[1]]$`MR-PRESSO results`$`Global Test`$Pvalue	[1] 0.096
GCST90032275+GCST90274773	[[1]]$`MR-PRESSO results`$`Global Test`$Pvalue	[1] 0.399
GCST90032275+GCST90274774	[[1]]$`MR-PRESSO results`$`Global Test`$Pvalue	[1] 0.608
GCST90032275+GCST90274780	[[1]]$`MR-PRESSO results`$`Global Test`$Pvalue	[1] 0.225
GCST90032275+GCST90274781	[[1]]$`MR-PRESSO results`$`Global Test`$Pvalue	[1] 0.515
GCST90032275+GCST90274813	[[1]]$`MR-PRESSO results`$`Global Test`$Pvalue	[1] 0.587
GCST90032275+GCST90274831	[[1]]$`MR-PRESSO results`$`Global Test`$Pvalue	[1] 0.604
GCST90032275+GCST90274835	[[1]]$`MR-PRESSO results`$`Global Test`$Pvalue	[1] 0.074
GCST90032279+GCST90274762	[[1]]$`MR-PRESSO results`$`Global Test`$Pvalue	[1] 0.31
GCST90032279+GCST90274773	[[1]]$`MR-PRESSO results`$`Global Test`$Pvalue	[1] 0.068
GCST90032279+GCST90274774	[[1]]$`MR-PRESSO results`$`Global Test`$Pvalue	[1] 0.585
GCST90032279+GCST90274780	[[1]]$`MR-PRESSO results`$`Global Test`$Pvalue	[1] 0.058
GCST90032279+GCST90274781	[[1]]$`MR-PRESSO results`$`Global Test`$Pvalue	[1] 0.449
GCST90032279+GCST90274813	[[1]]$`MR-PRESSO results`$`Global Test`$Pvalue	[1] 0.66

GCST90032279+GCST90274831	[[1]]$`MR-PRESSO results`$`Global Test`$Pvalue	[1] 0.251
GCST90032279+GCST90274835	[[1]]$`MR-PRESSO results`$`Global Test`$Pvalue	[1] 0.265
GCST90032290+GCST90274762	[[1]]$`MR-PRESSO results`$`Global Test`$Pvalue	[1] 0.889
GCST90032290+GCST90274773	[[1]]$`MR-PRESSO results`$`Global Test`$Pvalue	[1] 0.318
GCST90032290+GCST90274774	[[1]]$`MR-PRESSO results`$`Global Test`$Pvalue	[1] 0.521
GCST90032290+GCST90274780	[[1]]$`MR-PRESSO results`$`Global Test`$Pvalue	[1] 0.906
GCST90032290+GCST90274781	[[1]]$`MR-PRESSO results`$`Global Test`$Pvalue	[1] 0.501
GCST90032290+GCST90274813	[[1]]$`MR-PRESSO results`$`Global Test`$Pvalue	[1] 0.931
GCST90032290+GCST90274831	[[1]]$`MR-PRESSO results`$`Global Test`$Pvalue	[1] 0.49
GCST90032290+GCST90274835	[[1]]$`MR-PRESSO results`$`Global Test`$Pvalue	[1] 0.641
GCST90032293+GCST90274762	[[1]]$`MR-PRESSO results`$`Global Test`$Pvalue	[1] 0.326
GCST90032293+GCST90274773	[[1]]$`MR-PRESSO results`$`Global Test`$Pvalue	[1] 0.051
GCST90032293+GCST90274774	[[1]]$`MR-PRESSO results`$`Global Test`$Pvalue	[1] 0.719
GCST90032293+GCST90274780	[[1]]$`MR-PRESSO results`$`Global Test`$Pvalue	[1] 0.696
GCST90032293+GCST90274781	[[1]]$`MR-PRESSO results`$`Global Test`$Pvalue	[1] 0.105
GCST90032293+GCST90274813	[[1]]$`MR-PRESSO results`$`Global Test`$Pvalue	[1] 0.677
GCST90032293+GCST90274831	[[1]]$`MR-PRESSO results`$`Global Test`$Pvalue	[1] 0.701
GCST90032293+GCST90274835	[[1]]$`MR-PRESSO results`$`Global Test`$Pvalue	[1] 0.241
GCST90032298+GCST90274762	[[1]]$`MR-PRESSO results`$`Global Test`$Pvalue	[1] 0.404
GCST90032298+GCST90274773	[[1]]$`MR-PRESSO results`$`Global Test`$Pvalue	[1] 0.865
GCST90032298+GCST90274774	[[1]]$`MR-PRESSO results`$`Global Test`$Pvalue	[1] 0.424
GCST90032298+GCST90274780	[[1]]$`MR-PRESSO results`$`Global Test`$Pvalue	[1] 0.052
GCST90032298+GCST90274781	[[1]]$`MR-PRESSO results`$`Global Test`$Pvalue	[1] 0.233
GCST90032298+GCST90274813	[[1]]$`MR-PRESSO results`$`Global Test`$Pvalue	[1] 0.4
GCST90032298+GCST90274831	[[1]]$`MR-PRESSO results`$`Global Test`$Pvalue	[1] 0.231
GCST90032298+GCST90274835	[[1]]$`MR-PRESSO results`$`Global Test`$Pvalue	[1] 0.456
GCST90032302+GCST90274762	[[1]]$`MR-PRESSO results`$`Global Test`$Pvalue	[1] 0.161
GCST90032302+GCST90274773	[[1]]$`MR-PRESSO results`$`Global Test`$Pvalue	[1] 0.91
GCST90032302+GCST90274774	[[1]]$`MR-PRESSO results`$`Global Test`$Pvalue	[1] 0.834
GCST90032302+GCST90274780	[[1]]$`MR-PRESSO results`$`Global Test`$Pvalue	[1] 0.715

GCST90032302+GCST90274781	[[1]]$`MR-PRESSO results`$`Global Test`$Pvalue	[1] 0.412
GCST90032302+GCST90274813	[[1]]$`MR-PRESSO results`$`Global Test`$Pvalue	[1] 0.264
GCST90032302+GCST90274831	[[1]]$`MR-PRESSO results`$`Global Test`$Pvalue	[1] 0.639
GCST90032302+GCST90274835	[[1]]$`MR-PRESSO results`$`Global Test`$Pvalue	[1] 0.691
GCST90032319+GCST90274762	[[1]]$`MR-PRESSO results`$`Global Test`$Pvalue	[1] 0.355
GCST90032319+GCST90274773	[[1]]$`MR-PRESSO results`$`Global Test`$Pvalue	[1] 0.439
GCST90032319+GCST90274774	[[1]]$`MR-PRESSO results`$`Global Test`$Pvalue	[1] 0.148
GCST90032319+GCST90274780	[[1]]$`MR-PRESSO results`$`Global Test`$Pvalue	[1] 0.243
GCST90032319+GCST90274781	[[1]]$`MR-PRESSO results`$`Global Test`$Pvalue	[1] 0.249
GCST90032319+GCST90274813	[[1]]$`MR-PRESSO results`$`Global Test`$Pvalue	[1] 0.118
GCST90032319+GCST90274831	[[1]]$`MR-PRESSO results`$`Global Test`$Pvalue	[1] 0.054
GCST90032319+GCST90274835	[[1]]$`MR-PRESSO results`$`Global Test`$Pvalue	[1] 0.165
GCST90032327+GCST90274762	[[1]]$`MR-PRESSO results`$`Global Test`$Pvalue	[1] 0.613
GCST90032327+GCST90274773	[[1]]$`MR-PRESSO results`$`Global Test`$Pvalue	[1] 0.303
GCST90032327+GCST90274774	[[1]]$`MR-PRESSO results`$`Global Test`$Pvalue	[1] 0.335
GCST90032327+GCST90274780	[[1]]$`MR-PRESSO results`$`Global Test`$Pvalue	[1] 0.292
GCST90032327+GCST90274781	[[1]]$`MR-PRESSO results`$`Global Test`$Pvalue	[1] 0.256
GCST90032327+GCST90274813	[[1]]$`MR-PRESSO results`$`Global Test`$Pvalue	[1] 0.368
GCST90032327+GCST90274831	[[1]]$`MR-PRESSO results`$`Global Test`$Pvalue	[1] 0.6
GCST90032327+GCST90274835	[[1]]$`MR-PRESSO results`$`Global Test`$Pvalue	[1] 0.57
GCST90032335+GCST90274762	[[1]]$`MR-PRESSO results`$`Global Test`$Pvalue	[1] 0.298
GCST90032335+GCST90274773	[[1]]$`MR-PRESSO results`$`Global Test`$Pvalue	[1] 0.365
GCST90032335+GCST90274774	[[1]]$`MR-PRESSO results`$`Global Test`$Pvalue	[1] 0.002
GCST90032335+GCST90274780	[[1]]$`MR-PRESSO results`$`Global Test`$Pvalue	[1] 0.281
GCST90032335+GCST90274781	[[1]]$`MR-PRESSO results`$`Global Test`$Pvalue	[1] 0.511
GCST90032335+GCST90274813	[[1]]$`MR-PRESSO results`$`Global Test`$Pvalue	[1] 0.017
GCST90032335+GCST90274831	[[1]]$`MR-PRESSO results`$`Global Test`$Pvalue	[1] 0.217
GCST90032335+GCST90274835	[[1]]$`MR-PRESSO results`$`Global Test`$Pvalue	[1] 0.143
GCST90032373+GCST90274762	[[1]]$`MR-PRESSO results`$`Global Test`$Pvalue	[1] 0.705
GCST90032373+GCST90274773	[[1]]$`MR-PRESSO results`$`Global Test`$Pvalue	[1] 0.65

GCST90032373+GCST90274774	[[1]]$`MR-PRESSO results`$`Global Test`$Pvalue	[1] 0.587
GCST90032373+GCST90274780	[[1]]$`MR-PRESSO results`$`Global Test`$Pvalue	[1] 0.905
GCST90032373+GCST90274781	[[1]]$`MR-PRESSO results`$`Global Test`$Pvalue	[1] 0.884
GCST90032373+GCST90274813	[[1]]$`MR-PRESSO results`$`Global Test`$Pvalue	[1] 0.517
GCST90032373+GCST90274831	[[1]]$`MR-PRESSO results`$`Global Test`$Pvalue	[1] 0.554
GCST90032373+GCST90274835	[[1]]$`MR-PRESSO results`$`Global Test`$Pvalue	[1] 0.992
GCST90032423+GCST90274762	[[1]]$`MR-PRESSO results`$`Global Test`$Pvalue	[1] 0.295
GCST90032423+GCST90274773	[[1]]$`MR-PRESSO results`$`Global Test`$Pvalue	[1] 0.803
GCST90032423+GCST90274774	[[1]]$`MR-PRESSO results`$`Global Test`$Pvalue	[1] 0.416
GCST90032423+GCST90274780	[[1]]$`MR-PRESSO results`$`Global Test`$Pvalue	[1] 0.292
GCST90032423+GCST90274781	[[1]]$`MR-PRESSO results`$`Global Test`$Pvalue	[1] 0.552
GCST90032423+GCST90274813	[[1]]$`MR-PRESSO results`$`Global Test`$Pvalue	[1] 0.659
GCST90032423+GCST90274831	[[1]]$`MR-PRESSO results`$`Global Test`$Pvalue	[1] 0.587
GCST90032423+GCST90274835	[[1]]$`MR-PRESSO results`$`Global Test`$Pvalue	[1] 0.847
GCST90032435+GCST90274762	[[1]]$`MR-PRESSO results`$`Global Test`$Pvalue	[1] 0.411
GCST90032435+GCST90274773	[[1]]$`MR-PRESSO results`$`Global Test`$Pvalue	[1] 0.989
GCST90032435+GCST90274774	[[1]]$`MR-PRESSO results`$`Global Test`$Pvalue	[1] 0.933
GCST90032435+GCST90274780	[[1]]$`MR-PRESSO results`$`Global Test`$Pvalue	[1] 0.851
GCST90032435+GCST90274781	[[1]]$`MR-PRESSO results`$`Global Test`$Pvalue	[1] 0.694
GCST90032435+GCST90274813	[[1]]$`MR-PRESSO results`$`Global Test`$Pvalue	[1] 0.494
GCST90032435+GCST90274831	[[1]]$`MR-PRESSO results`$`Global Test`$Pvalue	[1] 0.555
GCST90032435+GCST90274835	[[1]]$`MR-PRESSO results`$`Global Test`$Pvalue	[1] 0.842
GCST90032443+GCST90274762	[[1]]$`MR-PRESSO results`$`Global Test`$Pvalue	[1] 0.722
GCST90032443+GCST90274773	[[1]]$`MR-PRESSO results`$`Global Test`$Pvalue	[1] 0.174
GCST90032443+GCST90274774	[[1]]$`MR-PRESSO results`$`Global Test`$Pvalue	[1] 0.724
GCST90032443+GCST90274780	[[1]]$`MR-PRESSO results`$`Global Test`$Pvalue	[1] 0.126
GCST90032443+GCST90274781	[[1]]$`MR-PRESSO results`$`Global Test`$Pvalue	[1] 0.206
GCST90032443+GCST90274813	[[1]]$`MR-PRESSO results`$`Global Test`$Pvalue	[1] 0.033
GCST90032443+GCST90274831	[[1]]$`MR-PRESSO results`$`Global Test`$Pvalue	[1] 0.52
GCST90032443+GCST90274835	[[1]]$`MR-PRESSO results`$`Global Test`$Pvalue	[1] 0.4

GCST90032455+GCST90274762	[[1]]$`MR-PRESSO results`$`Global Test`$Pvalue	[1] 0.845
GCST90032455+GCST90274773	[[1]]$`MR-PRESSO results`$`Global Test`$Pvalue	[1] 0.315
GCST90032455+GCST90274774	[[1]]$`MR-PRESSO results`$`Global Test`$Pvalue	[1] 0.854
GCST90032455+GCST90274780	[[1]]$`MR-PRESSO results`$`Global Test`$Pvalue	[1] 0.893
GCST90032455+GCST90274781	[[1]]$`MR-PRESSO results`$`Global Test`$Pvalue	[1] 0.46
GCST90032455+GCST90274813	[[1]]$`MR-PRESSO results`$`Global Test`$Pvalue	[1] 0.339
GCST90032455+GCST90274831	[[1]]$`MR-PRESSO results`$`Global Test`$Pvalue	[1] 0.705
GCST90032455+GCST90274835	[[1]]$`MR-PRESSO results`$`Global Test`$Pvalue	[1] 0.775
GCST90032459+GCST90274762	[[1]]$`MR-PRESSO results`$`Global Test`$Pvalue	[1] 0.56
GCST90032459+GCST90274773	[[1]]$`MR-PRESSO results`$`Global Test`$Pvalue	[1] 0.762
GCST90032459+GCST90274774	[[1]]$`MR-PRESSO results`$`Global Test`$Pvalue	[1] 0.625
GCST90032459+GCST90274780	[[1]]$`MR-PRESSO results`$`Global Test`$Pvalue	[1] 0.869
GCST90032459+GCST90274781	[[1]]$`MR-PRESSO results`$`Global Test`$Pvalue	[1] 0.876
GCST90032459+GCST90274813	[[1]]$`MR-PRESSO results`$`Global Test`$Pvalue	[1] 0.876
GCST90032459+GCST90274831	[[1]]$`MR-PRESSO results`$`Global Test`$Pvalue	[1] 0.326
GCST90032459+GCST90274835	[[1]]$`MR-PRESSO results`$`Global Test`$Pvalue	[1] 0.495
GCST90032491+GCST90274762	[[1]]$`MR-PRESSO results`$`Global Test`$Pvalue	[1] 0.839
GCST90032491+GCST90274773	[[1]]$`MR-PRESSO results`$`Global Test`$Pvalue	[1] 0.205
GCST90032491+GCST90274774	[[1]]$`MR-PRESSO results`$`Global Test`$Pvalue	[1] 0.735
GCST90032491+GCST90274780	[[1]]$`MR-PRESSO results`$`Global Test`$Pvalue	[1] 0.027
GCST90032491+GCST90274781	[[1]]$`MR-PRESSO results`$`Global Test`$Pvalue	[1] 0.316
GCST90032491+GCST90274813	[[1]]$`MR-PRESSO results`$`Global Test`$Pvalue	[1] 0.443
GCST90032491+GCST90274831	[[1]]$`MR-PRESSO results`$`Global Test`$Pvalue	[1] 0.018
GCST90032491+GCST90274835	[[1]]$`MR-PRESSO results`$`Global Test`$Pvalue	[1] 0.625
GCST90032494+GCST90274762	[[1]]$`MR-PRESSO results`$`Global Test`$Pvalue	[1] 0.367
GCST90032494+GCST90274773	[[1]]$`MR-PRESSO results`$`Global Test`$Pvalue	[1] 0.874
GCST90032494+GCST90274774	[[1]]$`MR-PRESSO results`$`Global Test`$Pvalue	[1] 0.234
GCST90032494+GCST90274780	[[1]]$`MR-PRESSO results`$`Global Test`$Pvalue	[1] 0.628
GCST90032494+GCST90274781	[[1]]$`MR-PRESSO results`$`Global Test`$Pvalue	[1] 0.327
GCST90032494+GCST90274813	[[1]]$`MR-PRESSO results`$`Global Test`$Pvalue	[1] 0.565

GCST90032494+GCST90274831	[[1]]$`MR-PRESSO results`$`Global Test`$Pvalue	[1] 0.446
GCST90032494+GCST90274835	[[1]]$`MR-PRESSO results`$`Global Test`$Pvalue	[1] 0.631
GCST90032503+GCST90274762	[[1]]$`MR-PRESSO results`$`Global Test`$Pvalue	[1] 0.977
GCST90032503+GCST90274773	[[1]]$`MR-PRESSO results`$`Global Test`$Pvalue	[1] 0.835
GCST90032503+GCST90274774	[[1]]$`MR-PRESSO results`$`Global Test`$Pvalue	[1] 0.609
GCST90032503+GCST90274780	[[1]]$`MR-PRESSO results`$`Global Test`$Pvalue	[1] 0.549
GCST90032503+GCST90274781	[[1]]$`MR-PRESSO results`$`Global Test`$Pvalue	[1] 0.971
GCST90032503+GCST90274813	[[1]]$`MR-PRESSO results`$`Global Test`$Pvalue	[1] 0.954
GCST90032503+GCST90274831	[[1]]$`MR-PRESSO results`$`Global Test`$Pvalue	[1] 0.269
GCST90032503+GCST90274835	[[1]]$`MR-PRESSO results`$`Global Test`$Pvalue	[1] 0.572
GCST90032504+GCST90274762	[[1]]$`MR-PRESSO results`$`Global Test`$Pvalue	[1] 0.757
GCST90032504+GCST90274773	[[1]]$`MR-PRESSO results`$`Global Test`$Pvalue	[1] 0.123
GCST90032504+GCST90274774	[[1]]$`MR-PRESSO results`$`Global Test`$Pvalue	[1] 0.874
GCST90032504+GCST90274780	[[1]]$`MR-PRESSO results`$`Global Test`$Pvalue	[1] 0.768
GCST90032504+GCST90274781	[[1]]$`MR-PRESSO results`$`Global Test`$Pvalue	[1] 0.524
GCST90032504+GCST90274813	[[1]]$`MR-PRESSO results`$`Global Test`$Pvalue	[1] 0.817
GCST90032504+GCST90274831	[[1]]$`MR-PRESSO results`$`Global Test`$Pvalue	[1] 0.569
GCST90032504+GCST90274835	[[1]]$`MR-PRESSO results`$`Global Test`$Pvalue	[1] 0.657
GCST90032506+GCST90274762	[[1]]$`MR-PRESSO results`$`Global Test`$Pvalue	[1] 0.121
GCST90032506+GCST90274773	[[1]]$`MR-PRESSO results`$`Global Test`$Pvalue	[1] 0.095
GCST90032506+GCST90274774	[[1]]$`MR-PRESSO results`$`Global Test`$Pvalue	[1] 0.134
GCST90032506+GCST90274780	[[1]]$`MR-PRESSO results`$`Global Test`$Pvalue	[1] 0.145
GCST90032506+GCST90274781	[[1]]$`MR-PRESSO results`$`Global Test`$Pvalue	[1] 0.045
GCST90032506+GCST90274813	[[1]]$`MR-PRESSO results`$`Global Test`$Pvalue	[1] 0.406
GCST90032506+GCST90274831	[[1]]$`MR-PRESSO results`$`Global Test`$Pvalue	[1] 0.84
GCST90032506+GCST90274835	[[1]]$`MR-PRESSO results`$`Global Test`$Pvalue	[1] 0.579
GCST90032550+GCST90274762	[[1]]$`MR-PRESSO results`$`Global Test`$Pvalue	[1] 0.107
GCST90032550+GCST90274773	[[1]]$`MR-PRESSO results`$`Global Test`$Pvalue	[1] 0.068
GCST90032550+GCST90274774	[[1]]$`MR-PRESSO results`$`Global Test`$Pvalue	[1] 0.447
GCST90032550+GCST90274780	[[1]]$`MR-PRESSO results`$`Global Test`$Pvalue	[1] 0.171

GCST90032550+GCST90274781	[[1]]$`MR-PRESSO results`$`Global Test`$Pvalue	[1] 0.003
GCST90032550+GCST90274813	[[1]]$`MR-PRESSO results`$`Global Test`$Pvalue	[1] 0.66
GCST90032550+GCST90274831	[[1]]$`MR-PRESSO results`$`Global Test`$Pvalue	[1] 0.031
GCST90032550+GCST90274835	[[1]]$`MR-PRESSO results`$`Global Test`$Pvalue	[1] 0.109
GCST90032588+GCST90274762	[[1]]$`MR-PRESSO results`$`Global Test`$Pvalue	[1] 0.333
GCST90032588+GCST90274773	[[1]]$`MR-PRESSO results`$`Global Test`$Pvalue	[1] 0.176
GCST90032588+GCST90274774	[[1]]$`MR-PRESSO results`$`Global Test`$Pvalue	[1] 0.304
GCST90032588+GCST90274780	[[1]]$`MR-PRESSO results`$`Global Test`$Pvalue	[1] 0.074
GCST90032588+GCST90274781	[[1]]$`MR-PRESSO results`$`Global Test`$Pvalue	[1] 0.842
GCST90032588+GCST90274813	[[1]]$`MR-PRESSO results`$`Global Test`$Pvalue	[1] 0.374
GCST90032588+GCST90274831	[[1]]$`MR-PRESSO results`$`Global Test`$Pvalue	[1] 0.552
GCST90032588+GCST90274835	[[1]]$`MR-PRESSO results`$`Global Test`$Pvalue	[1] 0.926
GCST90032641+GCST90274762	[[1]]$`MR-PRESSO results`$`Global Test`$Pvalue	[1] 0.603
GCST90032641+GCST90274773	[[1]]$`MR-PRESSO results`$`Global Test`$Pvalue	[1] 0.515
GCST90032641+GCST90274774	[[1]]$`MR-PRESSO results`$`Global Test`$Pvalue	[1] 0.798
GCST90032641+GCST90274780	[[1]]$`MR-PRESSO results`$`Global Test`$Pvalue	[1] 0.333
GCST90032641+GCST90274781	[[1]]$`MR-PRESSO results`$`Global Test`$Pvalue	[1] 0.067
GCST90032641+GCST90274813	[[1]]$`MR-PRESSO results`$`Global Test`$Pvalue	[1] 0.77
GCST90032641+GCST90274831	[[1]]$`MR-PRESSO results`$`Global Test`$Pvalue	[1] 0.402
GCST90032641+GCST90274835	[[1]]$`MR-PRESSO results`$`Global Test`$Pvalue	[1] 0.125
GCST90032644+GCST90274762	[[1]]$`MR-PRESSO results`$`Global Test`$Pvalue	[1] 0.665
GCST90032644+GCST90274773	[[1]]$`MR-PRESSO results`$`Global Test`$Pvalue	[1] 0.575
GCST90032644+GCST90274774	[[1]]$`MR-PRESSO results`$`Global Test`$Pvalue	[1] 0.972
GCST90032644+GCST90274780	[[1]]$`MR-PRESSO results`$`Global Test`$Pvalue	[1] 0.307
GCST90032644+GCST90274781	[[1]]$`MR-PRESSO results`$`Global Test`$Pvalue	[1] 0.988
GCST90032644+GCST90274813	[[1]]$`MR-PRESSO results`$`Global Test`$Pvalue	[1] 0.462
GCST90032644+GCST90274831	[[1]]$`MR-PRESSO results`$`Global Test`$Pvalue	[1] 0.67
GCST90032644+GCST90274835	[[1]]$`MR-PRESSO results`$`Global Test`$Pvalue	[1] 0.215
